# A mouse model of Bardet-Biedl Syndrome has impaired fear memory, which is rescued by lithium treatment

**DOI:** 10.1371/journal.pgen.1009484

**Published:** 2021-04-22

**Authors:** Thomas K. Pak, Calvin S. Carter, Qihong Zhang, Sunny C. Huang, Charles Searby, Ying Hsu, Rebecca J. Taugher, Tim Vogel, Christopher C. Cychosz, Rachel Genova, Nina N. Moreira, Hanna Stevens, John A. Wemmie, Andrew A. Pieper, Kai Wang, Val C. Sheffield

**Affiliations:** 1 Medical Scientist Training Program, Roy J. and Lucille A. Carver College of Medicine, University of Iowa, Iowa City, Iowa, United States of America; 2 Neuroscience Program, Roy J. and Lucille A. Carver College of Medicine, University of Iowa, Iowa City, Iowa, United States of America; 3 Department of Pediatrics, Roy J. and Lucille A. Carver College of Medicine, University of Iowa, Iowa City, Iowa, United States of America; 4 Department of Psychiatry, Roy J. and Lucille A. Carver College of Medicine, University of Iowa, Iowa City, Iowa, United States of America; 5 Department of Veterans Affairs Medical Center, Iowa City, Iowa, United States of America; 6 Department of Orthopedics, Roy J. and Lucille A. Carver College of Medicine, University of Iowa, Iowa City, Iowa, United States of America; 7 Department of Obstetrics and Gynecology, Roy J. and Lucille A. Carver College of Medicine, University of Iowa, Iowa City, Iowa, United States of America; 8 Department of Molecular Physiology and Biophysics, Roy J. and Lucille A. Carver College of Medicine, University of Iowa, Iowa City, Iowa, United States of America; 9 Harrington Discovery Institute, University Hospitals Cleveland Medical Center, Cleveland, Ohio, United States of America; 10 Department of Psychiatry, Case Western Reserve University, Cleveland, Ohio, United States of America; 11 Geriatric Psychiatry, GRECC, Louis Stokes Cleveland VA Medical Center; Cleveland, Ohio, United States of America; 12 Institute for Transformative Molecular Medicine, School of Medicine, Case Western Reserve University, Cleveland, Ohio, United States of America; 13 Weill Cornell Autism Research Program, Weill Cornell Medicine of Cornell University, New York, United States of America; 14 Department of Neuroscience, Case Western Reserve University, School of Medicine, Cleveland, Ohio, United States of America; 15 Department of Biostatistics, College of Public Health, University of Iowa, Iowa City, Iowa, United States of America; HudsonAlpha Institute for Biotechnology, UNITED STATES

## Abstract

Primary cilia are microtubule-based organelles present on most cells that regulate many physiological processes, ranging from maintaining energy homeostasis to renal function. However, the role of these structures in the regulation of behavior remains unknown. To study the role of cilia in behavior, we employ mouse models of the human ciliopathy, Bardet-Biedl Syndrome (BBS). Here, we demonstrate that BBS mice have significant impairments in context fear conditioning, a form of associative learning. Moreover, we show that postnatal deletion of BBS gene function, as well as congenital deletion, specifically in the forebrain, impairs context fear conditioning. Analyses indicated that these behavioral impairments are not the result of impaired hippocampal long-term potentiation. However, our results indicate that these behavioral impairments are the result of impaired hippocampal neurogenesis. Two-week treatment with lithium chloride partially restores the proliferation of hippocampal neurons which leads to a rescue of context fear conditioning. Overall, our results identify a novel role of cilia genes in hippocampal neurogenesis and long-term context fear conditioning.

## Author summary

The primary cilium is a microtubule-based membranous projection on the cell that is involved in multiple physiological functions. Patients who have cilia dysfunction commonly have intellectual disability. However, it is not known how cilia affect learning and memory. Studying mouse models of a cilia-based intellectual disability can provide insight into learning and memory. One such cilia-based intellectual disability is Bardet-Biedl Syndrome (BBS), which is caused by homozygous and compound heterozygous mutations of BBS genes. We found that a mouse model of BBS (*Bbs1*^*M390R/M390R*^ mice) has learning and memory defects. In addition, we found that other mouse models of BBS have similar learning and memory defects. These BBS mouse models have difficulty associating an environment with a painful stimulus, a task designed to test context fear memory. This type of memory involves the brain hippocampus. This brain region produces new cells even into adulthood. The rate of new cell production in the hippocampus is important for learning and memory. *Bbs1*^*M390R/M390R*^ mice have decreased cell production in the hippocampus. Treating *Bbs1*^*M390R/M390R*^ mice with a compound (lithium) that increases cell production in the hippocampus improved the learning and memory deficits. Our results demonstrate a potential role for cilia in learning and memory, and indicate that lithium is a potential treatment, requiring further study, for the intellectual disability phenotype of BBS.

## Introduction

Intellectual disability (ID) is one of the most common neurodevelopmental disorders, affecting 1% of the global population [1,2]. Clinically, ID is characterized by a deficit in intellectual functioning and adaptive functioning [3]. There are limited pharmacological interventions for ID, partially due to a poor understanding of ID and its heterogeneous nature which can be attributed to a lack of animal models of ID [4,5]. There is an urgent need to develop animal models to improve our understanding of the pathophysiological mechanisms underlying this devastating condition.

Primary cilia are microtubule-based structures that extend from the surface of nearly all cells in the body, including neurons. Cilia play a role in maintaining energy homeostasis and facilitating physiological responses to sensory stimuli [6]. Patients with abnormal cilia, i.e. ciliopathies, frequently present with ID, suggesting that cilia play an important role in learning and memory, yet the mechanisms underlying the phenotype remain unknown [7]. Fortunately, there are robust mouse models of ciliopathies that recapitulate the primary features of these diseases. However, the regulation of behavioral responses remains not well understood. We reasoned that studying genetic ciliopathy mouse models can provide insights into the role of cilia in learning and memory. To this end, we employ mouse models of the human ciliopathy, Bardet-Biedl Syndrome (BBS), which presents clinically with intellectual disability [8] in order to investigate the role of cilia in learning and memory. BBS is a genetically heterogenous autosomal recessive ciliopathy with 22 known causative genes [9]. Clinical features of BBS include rod-cone dystrophy progressing to blindness, postaxial polydactyly, obesity, renal anomalies, and intellectual disability [10]. BBS proteins are involved in ciliary function. Eight BBS genes, specifically *BBS1*, *BBS2*, *BBS4*, *BBS5*, *BBS7*, *BBS8*, *BBS9*, and *BBS18* (*BBIP1*), encode the components of the BBSome [11,12], an octameric protein complex. BBS1 M390R is the most common BBS mutation[13]. The BBSome regulates ciliary trafficking of G-Protein Coupled Receptors (GPCR) including SMO[14], NPY2R [15], MCHR1 and SSTR3[16], and D1R [17]), as well as non-GPCRs (TRKB[18]). Three non-BBSome BBS proteins (BBS6, BBS10, and BBS12) form a complex that mediates the assembly of the BBSome [19]. BBS3 is a GTPase that is also involved in ciliary receptor trafficking[20].

We have developed mouse models of BBS that recapitulate the major phenotypes of BBS [21]. We focused on the use of *Bbs1*^M390R/M390R^ mice, harboring the most common human BBS mutation, as it recapitulates many of BBS phenotypes present in patients, including obesity, retinopathy, and decreased hippocampal volume[22,23]. Despite the phenotypic association between decreased hippocampal volume in patients and the known role of the hippocampus in learning and memory, the role of BBS in learning and memory is not well studied. Here, we investigate the role of these cilia genes in learning and memory using a fear conditioning paradigm.

Fear conditioning evaluates associative learning and involves pairing a neutral stimulus [conditioned stimulus (CS)], to an aversive stimulus [unconditioned stimulus (US)]. Fear conditioning is commonly used to understand the neurobiological mechanisms of ID as well as fear learning and memory in mice due to several advantages [24–27]. First, fear conditioning paradigms provide distinct insights into the neural correlates of learning and memory, for example, context or cue-dependent conditioning, which require contributions from different brain regions [28]. Second, the pairing of CS to US consistently elicits a measurable set of physiological and behavioral responses [29]. Third, fear conditioning allows for the delineation between short-term and long-term memory performance, depending on the time duration from training to testing. To assess short-term context fear conditioning, a one-hour interval between training and testing is utilized. To evaluate long-term context fear conditioning, an interval ≥ 24 hours is utilized [30–32]. Finally, fear conditioning is a form of passive learning, thus fear conditioning can be used in many strains of rodents, even with motor deficits that may complicate other learning assays [33].

Here, we report that *Bbs1*^M390R/M390R^ mice have impaired long-term context fear conditioning, but normal short-term context memory. In addition, we show that a BBSome mouse model, postnatal *Bbs8* deletion mice, as well as a mouse model with forebrain specific deletion of *Bbs1*, have impaired long-term context fear conditioning. These results bring clarity to the conflicting result of fear conditioning in *Bbs4* knockout mice [34,35]. We also show a novel role for the *Bbs1* gene in neural proliferation and neurogenesis in the hippocampus. Finally, we show that two-week treatment with lithium chloride rescues long-term context fear conditioning and partially rescues hippocampal neurogenesis in *Bbs1*^M390R/M390R^ mice. Overall, this study shows a molecular connection between primary cilia and learning and memory using mouse models of BBS. Our study encourages further research to explore lithium as a potential therapeutic agent for treating intellectual disability in BBS patients.

## Results

### *Bbs1*^*M390R/M390R*^ mice have impaired long-term fear conditioning

**Fig 1:**
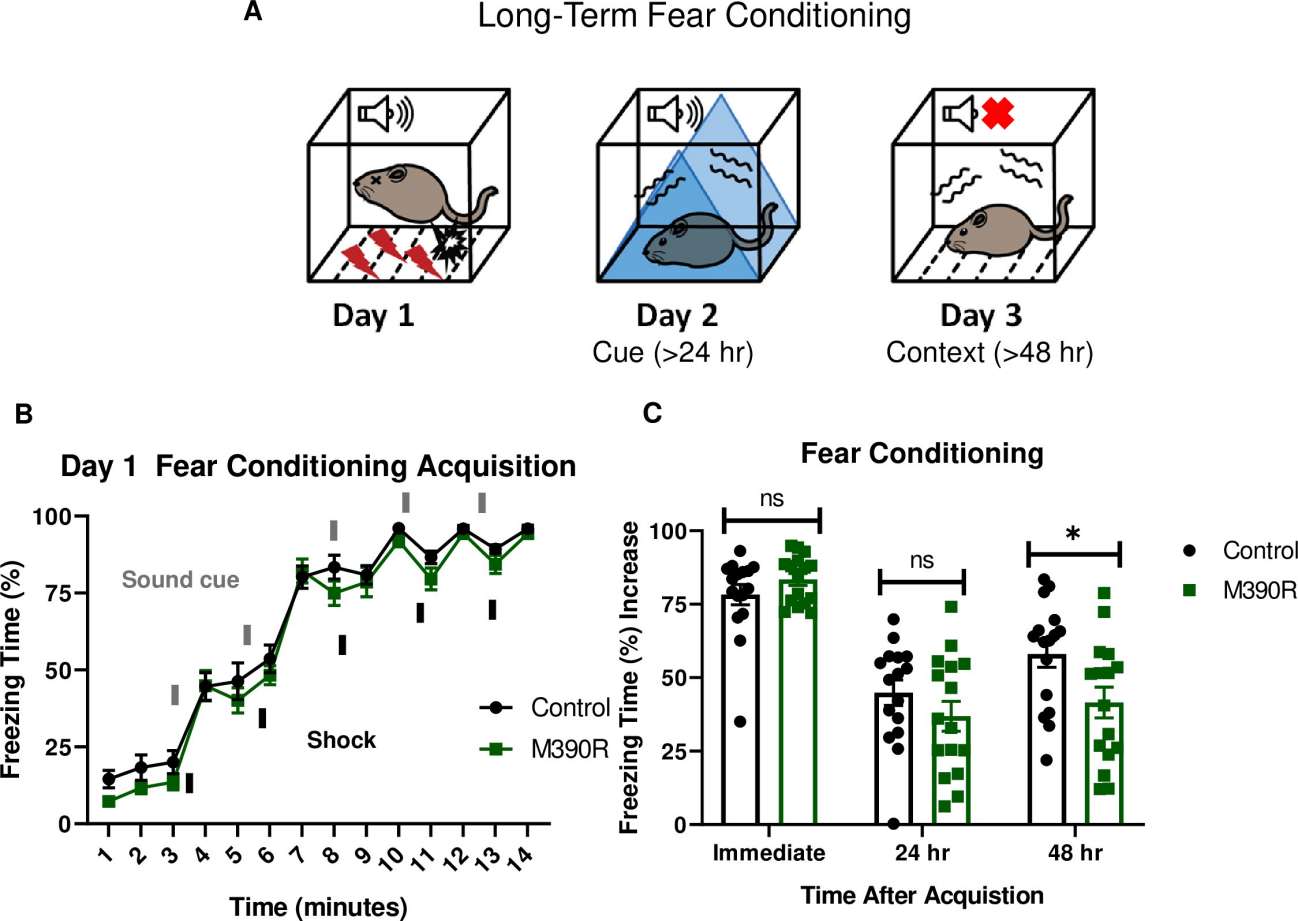
*Bbs1*^*M390R/M390R*^ mice have impaired long-term context fear conditioning. A.) Schematic diagram of the delay fear conditioning procedure. On the first day, a mouse is placed in a chamber, and a sound is paired with a shock multiple times. On the second day, a mouse is placed in an altered chamber. The chamber is triangle shaped (represented by the blue triangle) with a smooth floor, and a sound is given to test long-term cue fear conditioning. On the third day, the mouse is placed back in the same chamber (context) as day 1, without a sound cue. This set up is used to test long-term context fear conditioning. B.) Day 1 acquisition between the control mice (n = 16) and the *Bbs1*^*M390R/M390R*^ mice (n = 16) for long-term fear conditioning differed significantly (2-way ANOVA, time X genotype, F (13, 420) = 0.4488, P = 0.950, time, F (13, 420) = 179.8, P<0.0001, genotype, F (1, 420) = 11.46, P = 0.0008). The thick lines above the curve indicate when the sound cue was given, and the thick lines below the curve indicate when the shock was given. C.) The immediate fear conditioning indicates training to the day 1 fear conditioning. The immediate fear conditioning is measured as the difference of the freezing time (%) just before conditioning (first three minutes) and just after conditioning (last minute). The immediate fear conditioning did not differ significantly between the control mice (n = 16) and *Bbs1*^*M390/M390R*^ mice (n = 16) used for long-term fear conditioning (Welch’s t-test, P = 0.2107). The post 24 hr fear conditioning represents cue fear conditioning, and is portrayed as Day 2 on the schematic diagram. The 24 hr fear conditioning (cue) is measured as the difference of the freezing time (%) before the tone (cue) on day 2 and during the tone (cue) on day 2. The 24 hr fear conditioning (cue) did not differ significantly between the control mice (n = 16) and the *Bbs1*^*M390R/M390R*^ mice (n = 16) (Welch’s t-test, P = 0.2414). The post 48 hr fear conditioning represents context fear conditioning, and is portrayed as Day 3 on the schematic diagram. The 48 hr fear conditioning is measured as the difference of the freezing time (%) just before conditioning (first three minutes of day 1) and during the context on day 3. The 48 hr fear conditioning (context) between the control mice (n = 16) and the *Bbs1*^*M390R/M390R*^ mice (n = 16) differed significantly (Welch’s t-test, P = 0.0240). control mice = *Bbs1*^*M390R/+*^ mice, M390R = *Bbs1*^*M390R/M390R*^ mice, hr = hour, ns = not significant, * P< 0.05.

To study the learning and memory in BBS, we employed mouse models of the most common human BBS mutation, *Bbs1*^*M390R/M390R*^. Learning was evaluated using a three-day delay fear conditioning paradigm, which tests for long-term association memory (Fig 1A). Controls were littermate heterozygote or wild-type mice as there is no difference in fear conditioning between these animals (S1 Fig). The first day of fear conditioning is the acquisition phase where a sound cue is paired with a shock stimulus multiple times. Both *Bbs1*^*M390R/M390R*^ mice and their littermate controls showed increased freezing behavior following a shock stimulus, indicating that BBS mice exhibit a normal physiological response to an aversive stimulus (Fig 1B). On day 2 (post 24 hours from training), mice were introduced into a novel environment to test cue (sound) dependent fear conditioning. We found no significant differences between *Bbs1*^*M390R/M390R*^ and control mice, indicating that BBS mice have intact cue dependent learning (Fig 1C). On day 3 (post 48 hours from training), mice were re-introduced back into the training environment to test context (environment) dependent learning. Remarkably, *Bbs1*^*M390R/M390R*^ mice showed a 28% reduction in freeze behavior in this environment relative to littermate controls (Fig 1C). A sex difference was not observed in control mice or *Bbs1*^*M390R/M390R*^ mice for acquisition (immediate fear conditioning), cue fear conditioning (24 hours after acquisition), and context fear conditioning (48 hours after acquisition) (S2A and S2B Fig). These findings reveal that BBS mice have context specific fear conditioning impairments.

Due to the pleiotropic nature of BBS, we tested for confounding factors that may underlie the striking impairments in fear conditioning observed in *Bbs1*^*M390R/M390R*^ mice. No hearing differences were observed between *Bbs1*^M390R/M390R^ mice and control mice based on Auditory Brainstem Response and hearing behavior (S3A and S3B Fig). No differences were observed in shock reactivity between *Bbs1*^*M390R/M390R*^ mice and control mice indicating a normal tactile response (S3C Fig). Moreover, we did not observe a difference in activity levels or sleep behavior between *Bbs1*^*M390R/M390R*^ mice and control mice (S3D and S3E Fig). These findings reveal that the impaired fear response is not due to a secondary effect of these sensory systems.

### *Bbs1*^*M390R/M390R*^ mice have normal short-term fear conditioning

**Fig 2:**
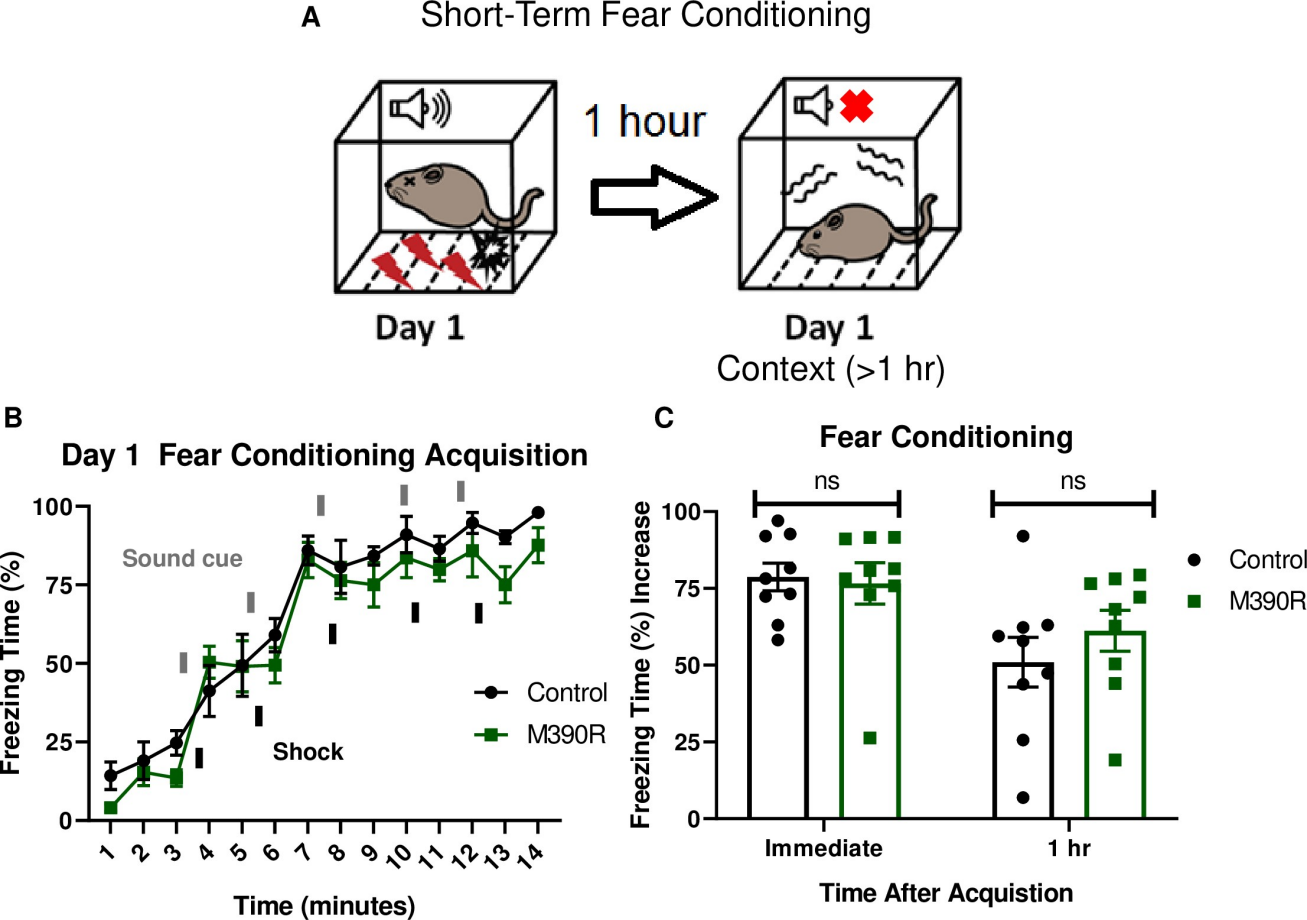
*Bbs1*^*M390R/M390R*^ mice have normal short-term context fear conditioning. A.) Schematic diagram of the one day delay fear conditioning procedure. On the first day, a mouse is placed in a chamber, and a sound is paired with a shock multiple times. One hour later, the mouse is placed back in the chamber, and freezing is measured for short-term context fear conditioning. B.) Day 1 acquisition between the control mice (n = 9) and the *Bbs1*^*M390R/M390R*^ mice (n = 9) used for the short-term fear conditioning differed significantly (2-way ANOVA, time X genotype, F (13, 224) = 0.5574, P = 0.8858, time, F (13, 224) = 56.19, P<0.0001, genotype, F (1, 224) = 9.369, P = 0.0025). The thick lines above the curve indicate when the sound cue was given, and the thick lines below the curve indicate when the shock was given. C.) The immediate fear conditioning indicates training to the day 1 fear conditioning. The immediate fear conditioning is measured as the difference of the freezing time (%) just before conditioning (first three minutes) and just after conditioning (last minute). The immediate fear conditioning did not differ significantly between control mice (n = 9) and *Bbs1*^*M390/M390R*^ mice (n = 9) used for the short-term fear conditioning (Welch’s t-test, P = 0.8004). The 1 hr fear conditioning represents short-term context fear conditioning. The 1 hr fear conditioning was measured as the difference of the freezing time (%) just before conditioning and 1 hour after conditioning. The day 1 fear conditioning for context between the control mice (n = 9) and the *Bbs1*^*M390R/M390R*^ mice (n = 9) did not reveal a significant difference (Welch’s t-test, P = 0.3436). control mice = *Bbs1*^*M390R/+*^ mice, M390R = *Bbs1*^*M390R/M390R*^ mice, hr = hour, ns = not significant.

We tested short-term fear conditioning in *Bbs1*^*M390R/M390R*^ mice to assess if the long-term memory deficit is due to short-term memory impairment. To test for short-term fear conditioning memory, we used a 1-day fear conditioning paradigm in which context fear conditioning was tested one hour after training (Fig 2A). Both the control mice (*Bbs1*^*M390R/+*^ mice) and *Bbs1*^*M390R/M390R*^ mice showed intact conditioning to shock (Fig 2B). In addition, there was no significant difference in short-term context memory performance between the control mice and *Bbs1*^*M390R/M390R*^ mice (Fig 2C). These results contrast with the differences in long-term context memory, which shows impaired performance in *Bbs1*^*M390R/M390R*^ mice compared to controls (Fig 1C). These findings indicate that *Bbs1*^*M390R/M390R*^ mice display specific impairments in long-term context fear conditioning. In addition, there is a possible confound that *Bbs1*^*M390R/M390R*^ mice express fear behavior differently from control mice, which would explain the long-term fear conditioning differences. However, the fact that there is not a difference in short-term context fear conditioning, which also utilizes the same fear behavior in long-term context fear conditioning, suggests there is not a difference in how the *Bbs1*^*M390R/M390R*^ mice express fear behaviors compared to control mice.

### Mice with postnatal deletion of *Bbs8* have impaired long-term context fear conditioning

**Fig 3:**
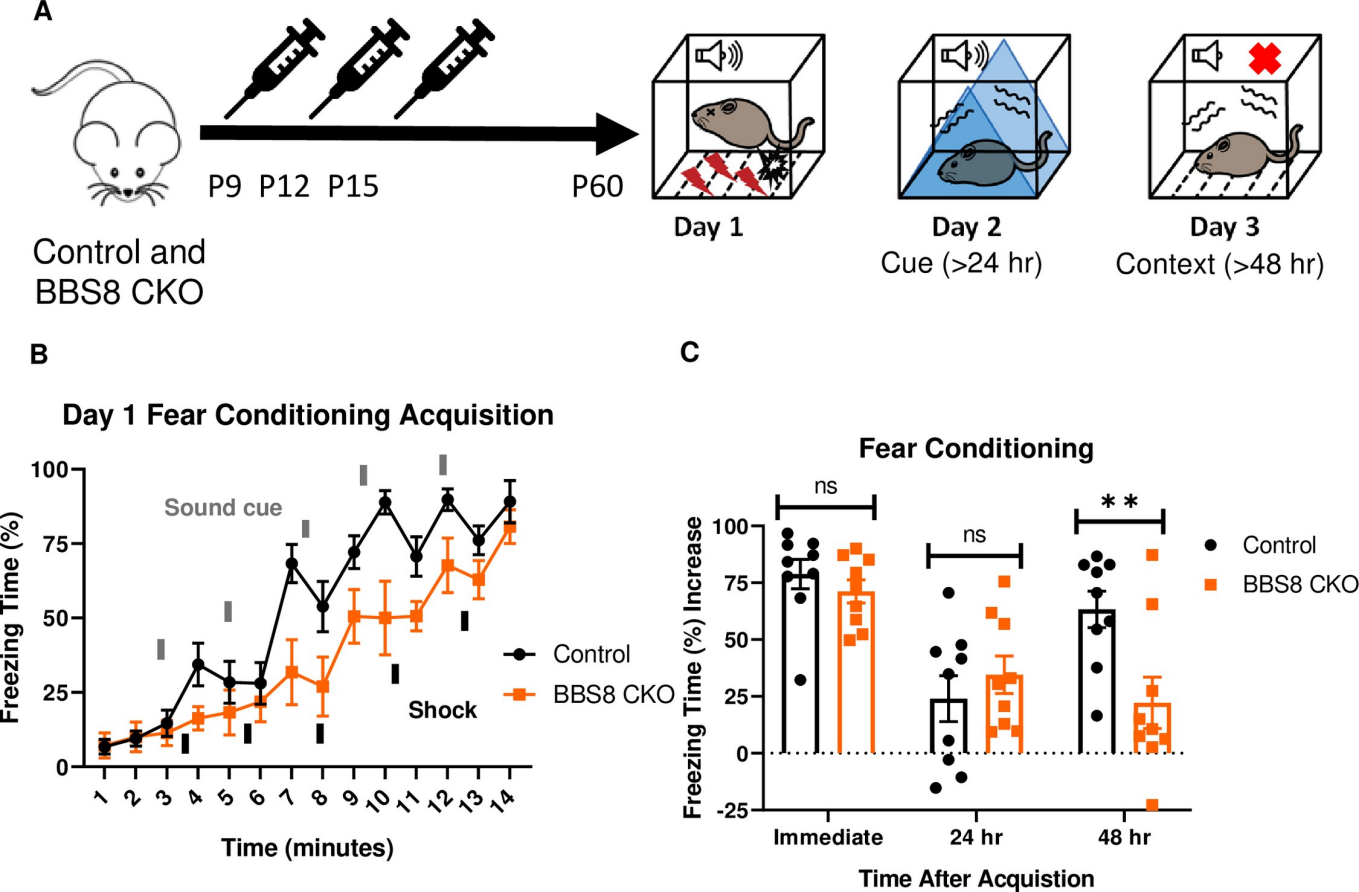
*Bbs8* is involved in long-term context fear conditioning postnatally. A.) Timeline of the tamoxifen I.P injections of the experimental mice, BBS8 CKO (*Bbs8*^flox/-^ and *Bbs8*^flox/flox^; *UBC-Cre*^*ERT2*^) and littermate control mice, Control (*Bbs8*^flox/-^ and *Bbs8*^flox/flox^; *UBC-Cre*^*ERT2*^*-*). To induce *Bbs8* deletion in BBS8 CKO mice, Tamoxifen was injected at P9, P12, and P15 (denoted by the syringe image). At 2 months of age, the mice were tested for long-term fear conditioning. The first day was the acquisition phase for fear conditioning, the second day was cue fear conditioning, and the third day was context fear conditioning. B.) Day 1 acquisition curve between the Control mice (n = 9) and BBS8 CKO mice (n = 9) differed significantly (2-way ANOVA, time X genotype, F (13, 224) = 1.721, p = 0.0579, time, F (13, 224) = 31.71, P<0.0001, genotype, F (1, 224) = 39.16, P<0.0001). The thick lines above the curve indicate when the sound cue was given, and the thick lines below the curve indicate when the shock was given. C.) The immediate fear conditioning indicates training to the day 1 fear conditioning. The immediate fear conditioning was measured as the difference of the freezing time (%) just before conditioning (first three minutes) and just after conditioning (last minute). The immediate fear conditioning did not differ significantly between the Control mice (n = 9) and BBS8 CKO mice (n = 9) used for long-term fear conditioning (Welch’s t-test, P = 0.3717). The post 24 hr fear conditioning represents cue fear conditioning, and is portrayed as Day 2 on the schematic diagram. The 24 hr fear conditioning (cue) was measured as the difference of the freezing time (%) before the tone (cue) on day 2 and during the tone (cue) on day 2. The 24 hr fear conditioning (cue) did not differ significantly between the Control mice (n = 9) and BBS8 CKO mice (n = 9) (Welch’s t-test, P = 0.4325). The post 48 hr fear conditioning represents context fear conditioning, and is portrayed as Day 3 on the schematic diagram. The 48 hr fear conditioning was measured as the difference of the freezing time (%) just before conditioning (first three minutes of day 1) and during the context on day 3. The 48 hr fear conditioning (context) between the Control mice (n = 9) and BBS8 CKO mice (n = 9) differed significantly (Welch’s t-test, P = 0.0099). Control *= Bbs8*^flox/-^ and *Bbs8*^flox/flox^*; UBC-Cre*^*ERT2*^*-* mice, BBS8 CKO = *Bbs8*^flox/-^ and *Bbs8*^flox/flox^; *UBC-Cre*^*ERT2*^ + mice, hr = hour, del = deletion, flx = flox, hr = hour, ns = not significant, ** P< 0.01.

We took advantage of another BBS mouse model to further explore the role of BBS genes (especially BBSome genes) in fear conditioning, specifically a tamoxifen inducible knockout mouse model of BBS8 [36]. BBS8, like BBS1, is a component of the BBSome [11]. Using *Bbs8* tamoxifen inducible knockout mice, we evaluated the temporal effects of BBS8 on fear conditioning (Fig 3A). For controls, we used littermates lacking *Cre*. Tamoxifen was administered to both groups of mice to control for possible effects of tamoxifen on behavior [37]. Following day 1 of fear conditioning, mice with *Bbs8* postnatally deleted, as well as control mice, were successfully conditioned to fear (Fig 3B). However, significant impairments in context but not cue fear conditioning were observed between conditional KO *Bbs8* mice and controls (Fig 3C). These results are similar to the results for *Bbs1*^*M390R/M390R*^ mice, indicating a role of the BBSome in mediating long-term context fear conditioning. Although, we observed differences in the acquisition curve of day 1 fear conditioning between conditional KO *Bbs8* mice and controls, no difference was found in immediate fear conditioning (Fig 3B and 3C). These results indicate that like *Bbs1*, *Bbs8* is involved in long-term context fear conditioning.

### Mice with preferential deletion of *Bbs1* in the forebrain have impaired long-term context fear conditioning

The forebrain contains brain regions involved in fear conditioning including the amygdala and hippocampus[28]. To explore whether the absence of normal BBS1 function in the forebrain is responsible for the fear conditioning impairment observed in *Bbs1*^*M390R/M390R*^ mice, we utilized a forebrain-specific *Bbs1* knockout mouse line developed by crossing a *Bbs1*^*flox/flox*^ conditional mouse line with a *Cre* line expressed in the forebrain (*Emx1*-*Cre* mice). The *Emx1*-*Cre* mice were generated and verified by Gorski et al. [38]. Using an Ai9 Cre reporter allele, we confirmed that the Cre is preferentially expressed in the forebrain (S4A and S4B Fig). We also confirmed that *Bbs1* is absent in the forebrain, but present in the hindbrain of *Bbs1*^*flox/flox*^, *Emx1-Cre*+ mice (S4C Fig), further confirming the specificity of *Cre* expression in *Emx1*-*Cre* mice.

**Fig 4:**
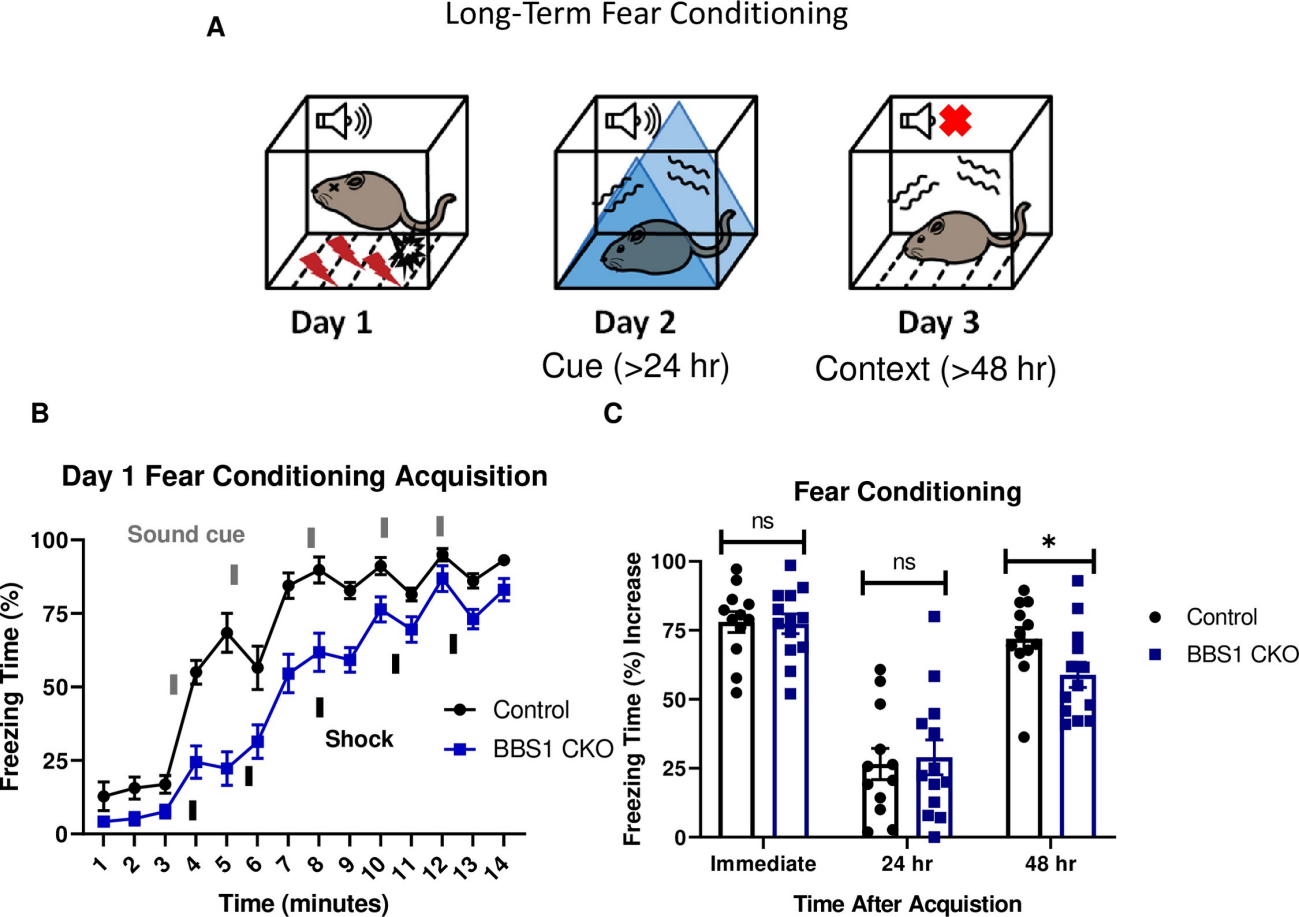
*Bbs1* in the forebrain is involved in long-term fear conditioning. A.) Schematic diagram of the three day delay fear conditioning procedure. On the first day, a mouse is placed in a chamber, and a sound is paired with a shock multiple time. On the second day, a mouse is placed in an altered chamber that is triangle shaped (represented by the blue triangle) with a smooth floor, and a sound is given to measure cue fear conditioning. On the third day, the mouse is placed back in the same chamber, and measured for freezing without sound. This gives the context fear conditioning. B.) Day 1 acquisition between the *Emx1-Cre* mice (control, mixed strain of C57BL/6 and 129/SVeV, n = 12) and the *Bbs1*^*flox/-*^; *Emx1-Cre* (BBS1 CKO, mixed strain of C57BL/6 and 129/SVeV, n = 13) differed significantly (2-way ANOVA, time X genotype, F (13, 322) = 3.483, P<0.0001, time, F (13, 322) = 93.95, P<0.0001, genotype, F (1, 322) = 137.5, P<0.0001). The thick lines above the curve indicate when the sound cue was given, and the thick lines below the curve indicate when the shock was given. C.) The immediate fear conditioning indicates training to the day 1 fear conditioning. The immediate fear conditioning is measured as the difference of the freezing time (%) just before conditioning (first three minutes) and just after conditioning (last minute). The immediate fear conditioning did not differ significantly between the control *Emx1-Cre* mice (n = 12) and *Bbs1*^*flox/-*^; *Emx1-Cre* (n = 13) used for the long-term fear conditioning (Welch’s t-test, P = 0.8999). The post 24 hr fear conditioning represents cue fear conditioning, and is portrayed as Day 2 on the schematic diagram. The 24 hr fear conditioning (cue) is measured as the difference of the freezing time (%) before the tone (cue) on day 2 and during the tone (cue) on day 2. The 24 hr fear conditioning (cue) did not differ significantly between the control *Emx1-Cre* mice (n = 12) and *Bbs1*^*flox/-*^; *Emx1-Cre* (n = 13) (Welch’s t-test, P = 0.7005). The post 48 hr fear conditioning represents context fear conditioning, and is portrayed as Day 3 on the schematic diagram. The 48 hr fear conditioning is measured as the difference of the freezing time (%) just before conditioning (first three minutes of day 1) and during the context on day 3. Day 3 fear conditioning for context between the control *Emx1-Cre* mice (n = 12) and *Bbs1*^*flox/-*^; *Emx1-Cre* (n = 13) differed significantly (Welch’s t-test, P = 0.0438, Mann-Whitney-Wilcoxon Test, P = 0.0398). control = *Emx1*-Cre mice, BBS1 CKO = *Bbs1*^*flox/-*^; *Emx1-Cre* mice, hr = hour, ns = not significant, * P< 0.05.

Control mice (*Emx1*-*Cre)* and forebrain specific *Bbs1* knockout mice (*Emx1*-*Cre*; *Bbs1*^*flox/-*^ mice) were fear conditioned using a three-day fear conditioning paradigm (Fig 4A). Acquisition of conditioning to shock was intact for both control mice and forebrain specific *Bbs1* knockout mice (Fig 4B). However, forebrain specific *Bbs1* knockout mice showed impaired context fear conditioning compared to controls (Fig 4C). Cue fear conditioning was observed to be intact for both knockout and control mice. These results indicate that BBS1 in the forebrain is required for contextual memory.

### *Bbs1*^M390R/M390R^ mice do not have impaired long-term potentiation

**Fig 5:**
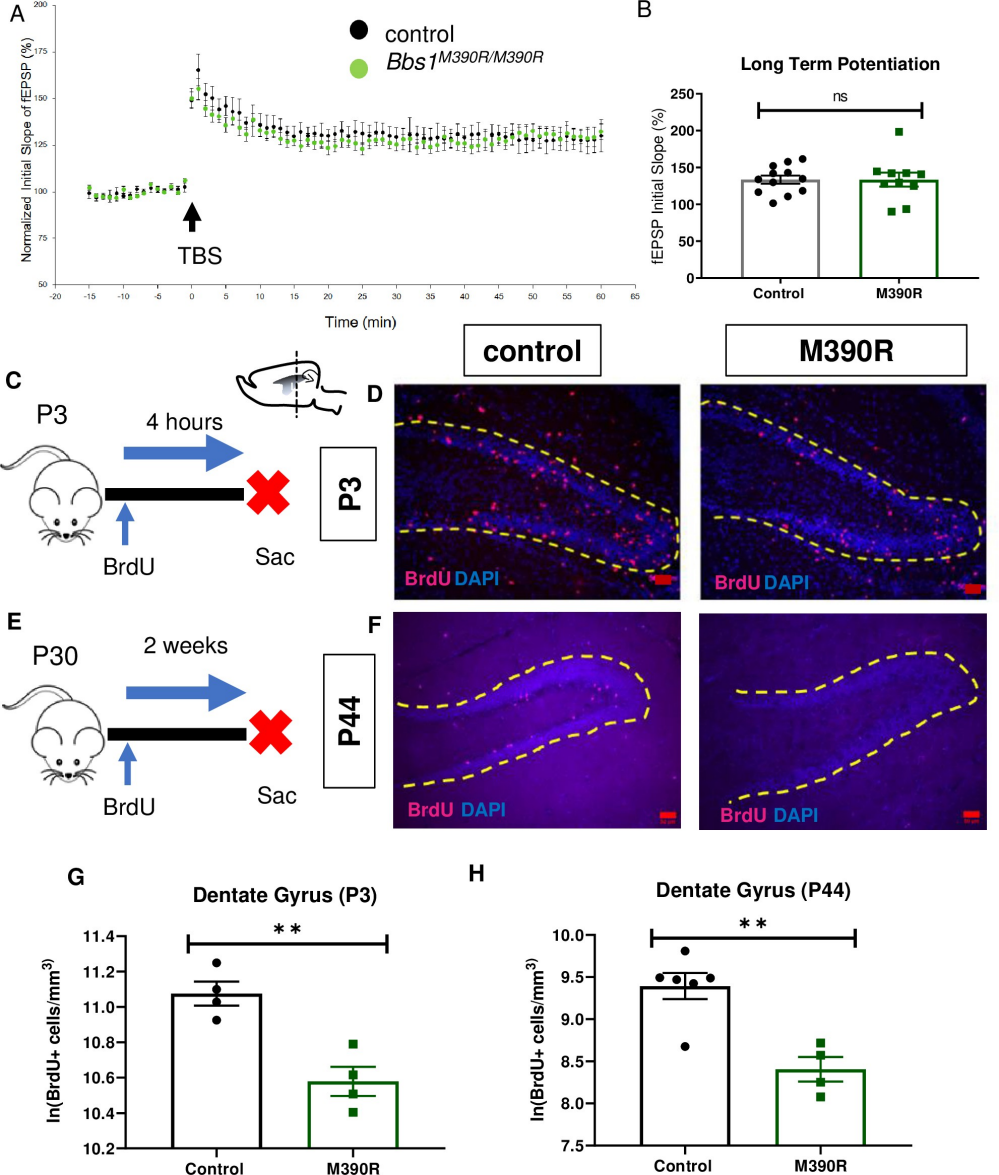
*Bbs1*^*M390R/M390R*^ mice have decreased hippocampal proliferation. A.) Normalized initial slope (%) recordings of field excitatory post synaptic potentials (fEPSP) in the hippocampal CA1 Schaffer-collateral pathway between 2 month male control mice (n = 17, 4 mice) and *Bbs1*^*M390R/M390R*^ mice (n = 16, 4 mice). LTP was induced by 12 theta burst stimulation (TBS). B.) The Long-Term Potentiation (average of last five minutes of normalized initial slope of fEPSP) in the hippocampal CA1 Schaffer-collateral pathway between 2 month male control mice (n = 17, 4 mice) and *Bbs1*^*M390R/M390R*^ mice (n = 16, 4 mice) did not differ significantly (Welch’s t-test, P = 0.8407). C.) Schematic diagram of the BrdU injections of postnatal day 3 (P3) mice. P3 mice were IP injected with 300mg/kg BrdU, and taken down four hours later. Sac = Sacrifice. D.) Inverted fluorescent microscope images of the P3 Dentate Gyrus. The sections were stained with Bromodeoxyruidine (BrdU) and counterstained with the nuclear marker, DAPI. BrdU immunostaining (red) and DAPI nuclear staining (blue). The yellow dotted line outlines the dentate gyrus. The Red Bar line represents 50μm.E.) Schematic diagram of the BrdU procedures for postnatal day 44 (P44) mice. At P30, mice were started on BrdU injections (2x50mg/kg) for five days. At P44, mice were taken down. Sac = Sacrifice. F.) Inverted fluorescent microscope images of the P44 Dentate Gyrus. The sections were stained with Bromodeoxyruidine (BrdU) and counterstained with the nuclear marker, DAPI. BrdU immunostaining (red) and DAPI nuclear staining (blue). The yellow dotted line outlines the dentate gyrus. The Red Bar line represents 50μm. G.) Decreased proliferation in the dentate gyrus of P3 *Bbs1*^*M390R/M390R*^ mice. The natural logarithm of BrdU+cell/mm^3^ in the dentate gyrus between the control mice (n = 4) and the *Bbs1*^*M390R/M390R*^ mice (n = 4) differed significantly (Welch’s t-test, P = 0.0038). H.) Decreased proliferation in the dentate gyrus of P44 *Bbs1*^*M390R/M390R*^ mice. The natural logarithm of BrdU+cell/mm^3^ in the dentate gyrus between the control mice (n = 6) and the *Bbs1*^*M390R/M390R*^ mice (n = 4) differed significantly (Welch’s t-test, P = 0.0018). control = *Bbs1*^*+/+*^, *Bbs1*^*M390R/+*^ mice, M390R = *Bbs1*^*M390R/M390R*^ mice, hr = hour, ns = not significant, ** P< 0.01.

Since *Bbs1*^*M390R/M390R*^ mice have impaired context fear conditioning, which is hippocampus dependent [28], we investigated hippocampal function in *Bbs1*^*M390R/M390R*^ mice. We evaluated long-term potentiation (LTP) in the CA1 region of the hippocampus because LTP is a neural correlate for long-term memory consolidation [39]. In addition, some mouse models with impaired context fear conditioning have impaired long-term potentiation (LTP) in CA1 of the hippocampus [30,40,41]. Despite the important role of LTP in fear conditioning, we did not observe a difference in LTP between control mice and *Bbs1*^*M390R/M390R*^ mice in CA1 of the hippocampus (Fig 5A and 5B). These results suggest that the observed impaired learning arises from causes other than impaired LTP.

### *Bbs1*^M390R/M390R^ mice have decreased hippocampal neurogenesis

Next, we sought to identify a potential cause of the defective long-term fear conditioning observed in BBS mice, It has been recently reported that BBS patients have decreased hippocampal volume which is thought to be a result of impaired neurogenesis [42]. Due to the known role that cilia play in mediating cell proliferation and hippocampal volume in patients [43,44], we hypothesized that defective hippocampal neurogenesis underlies the fear conditioning deficits in BBS mice. Therefore, we investigated hippocampal neurogenesis in *Bbs1*^*M390R/M390R*^ mice.

**Fig 6:**
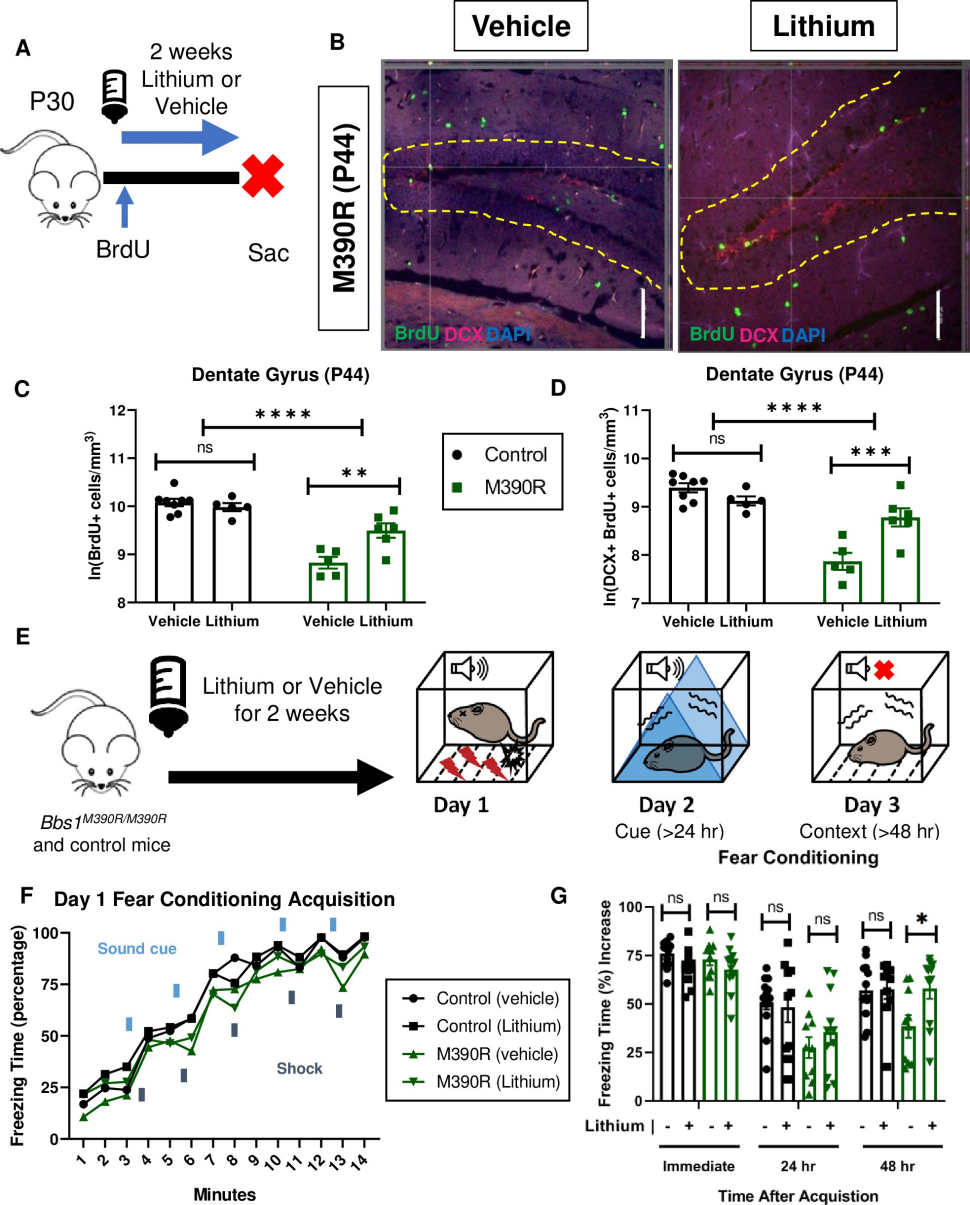
Chronic Lithium treatment rescued long-term context fear conditioning in *Bbs1*^*M390R/M390R*^ mice. A.) Schematic diagram of the Bromodeoxyuridine (BrdU) procedures for postnatal day 44 (P44) mice. At P30, mice were started on Lithium water (45mM) or continued with water (vehicle). Mice were also started on BrdU injections (2x50mg/kg) for five days. At P44, mice were taken down. Sac = Sacrifice. B.) Images of immunohistochemistry for neurogenesis of vehicle and lithium treated *Bbs1*^*M390R/M390R*^ mice. Z-stack, 20X, images of Dentate Gyrus at postnatal day 44 (P44). The tissue sections were stained with BrdU, Doublecortin (DCX), and counterstained with the nuclear marker DAPI. BrdU immunostaining (green), Doublecortin immunostaining (red) and DAPI nuclear staining (blue). The yellow dotted line outlines the dentate gyrus. The White Bar line represents 100 micrometers. C.) Proliferation in the dentate gyrus of P44 control and *Bbs1*^*M390R/M390R*^ mice. We assessed two factors, and found a significant interaction for genotype and treatment, and a difference in treatment and genotype (2-way ANOVA, treatment X genotype, F (1, 20) = 6.428, P = 0.0026, treatment, F (1, 20) = 1.569, P = 0.0177, genotype, F (1, 20) = 46.89, P<0.0001). A Sidak’s multiple comparisons test showed a significant difference in treatment for *Bbs1*^*M390R/M390R*^ mice (n = 5 Vehicle, n = 6 Lithium, P = 0.7875) but not for control mice (n = 8 Vehicle, n = 5 Lithium, P = 0.0010). D.) Neurogenesis in the dentate gyrus of P44 control and *Bbs1*^*M390R/M390R*^ mice. We assessed two factors, and found a significant interaction between genotype and treatment, and a difference in treatment and genotype (2-way ANOVA, treatment X genotype, F (1, 20) = 11.37, P = 0.0005, treatment, F (1, 20) = 0.0368, P = 0.5779, genotype, F (1, 20) = 29.54, P<0.0001). A Sidak’s multiple comparisons test showed a significant difference in treatment for *Bbs1*^*M390R/M390R*^ mice (n = 5 Vehicle, n = 6 Lithium, P = 0.0006) but not for control mice (n = 8 Vehicle, n = 5 Lithium, P = 0.3301). E.) Timeline of LiCl treatment. At 4–5 weeks of age, mice were treated with LiCl (45 mM) water or continued on water (vehicle). After two weeks of treatment, mice were tested on a 3 day fear conditioning set up. Day 1 is the training (acquisition phase) for fear conditioning. The second day is testing for cue fear conditioning. The third day is testing for context fear conditioning. F.) Day 1 fear conditioning acquisition between the vehicle treated control mice (n = 13) and *Bbs1*^*M390R/M390R*^ (45 mM) treated mice (n = 10), and lithium treated control mice (n = 9) and *Bbs1*^*M390R/M390R*^ (45 mM) treated mice (n = 11). Graph was presented without standard error. We found a significant difference in Treatment, Genotype, and Time (3-way ANOVA, Time x Genotype x Treatment, F (13, 560) = 0.2572, P = 0.9963; Genotype x Treatment, F (1, 560) = 0.4214, P = 0.5165; Time x Treatment, F (13, 560) = 1.338, P = 0.1860; Time x Genotype, F (13, 560) = 0.5960, P = 0.8582; Treatment, F (1, 560) = 5.475, P = 0.0196; Genotype, F (1, 560) = 39.48, P<0.0001; Time, F (13, 560) = 157.3, P<0.0001). The thick lines above the curve indicate when the sound cue was given, and the thick lines below the curve indicate when the shock was given. G.) The immediate fear conditioning indicates training to the day 1 fear conditioning. The immediate fear conditioning was measured as the freezing time (%) increase of the freezing time (%) just after conditioning (last minute) to the freezing time (%) just before conditioning (first three minutes). The immediate fear conditioning was not significantly different between the control mice given vehicle (n = 13) and control mice given LiCl water (n = 10) (Welch’s t-test, P = 0.0689) and did not significantly differ between the *Bbs1*^*M390R/M390R*^ mice given vehicle (n = 10) and *Bbs1*^*M390R/M390R*^ mice given LiCl water (45 mM) (n = 11) (Welch’s t-test, P = 0.2834). The post 24 hr fear conditioning represents the cue fear conditioning. The 24 hr fear conditioning (cue) was measured as the freezing time (%) increase of the freezing time (%) during the tone (cue, day 2) to the freezing time (%) before the tone (cue, day 2). The 24 hr fear conditioning (cue) was not significantly different between the control mice given vehicle (n = 13) and control mice given LiCl water (n = 10) (Welch’s t-test, P = 0.7483) and was not significantly different between the *Bbs1*^*M390R/M390R*^ mice given vehicle (n = 10) and *Bbs1*^*M390R/M390R*^ mice given LiCl water (45mM) (n = 11) (Welch’s t-test, P = 0.3625). The post 48 hr fear conditioning represents the context fear conditioning. The 48 hr fear conditioning was measured as the freezing time (%) increase of the freezing time (%) during the context on day 3 to the freezing time (%) just before conditioning (first three minutes of day 1). Day 3 fear conditioning for context was not significantly different between the control mice given vehicle (n = 13) and control mice given LiCl water (n = 10) (Welch’s t-test, P = 0.9285) but was significantly different between the *Bbs1*^*M390R/M390R*^ mice given vehicle (n = 10) and *Bbs1*^*M390R/M390R*^ mice given LiCl water (45mM) (n = 11) (Welch’s t-test, P = 0.0235). control = *Bbs1*^*+/+*^, *Bbs1*^*M390R/+*^ mice, M390R = *Bbs1*^*M390R/M390R*^ mice, hr = hour, ns = not significant, * P< 0.05, ** P< 0.01, ***P<0.001, ****P<0.0001.

To measure newly generated cells, we injected *Bbs1*^*M390R/M390R*^ and control mice with BrdU, a thymidine analog that is incorporated into replicating DNA to label proliferating cells (Fig 5C–5E). To measure proliferation in neonatal mice (P3), we sacrificed the mice four hours after BrdU injections. To measure cell survival and proliferation in young adult mice (P44), we sacrificed the mice two weeks after the start of BrdU injections. We also use this BrdU set up in young adult mice to measure neurogenesis. We found that both neonatal (P3) and young adult (P44) *Bbs1*^*M390R/M390R*^ mice displayed significant reductions in BrdU+ cells in the hippocampal dentate gyrus compared to controls (Fig 5D and 5F–5H). Moreover, young adult *Bbs1*^M390R/M390R^ mice also show fewer new neurons as determined by a reduced number of cells co-labeled for BrdU and Doublecortin, a marker for immature neurons (Fig 6B–6D)[45].

The role of the observed impairments in hippocampal neurogenesis in long-term context fear conditioning of *Bbs1*^*M390R/M390R*^ is unclear. To test the role of neurogenesis, we utilized a pharmacological modality to enhance hippocampal neurogenesis. Because impaired neurogenesis within the dentate gyrus is associated with long-term memory deficits, we reasoned that rescue of impaired neurogenesis could improve fear conditioning impairments. To this end, we chose lithium due to its previous use as an agent to improve neurogenesis and hippocampal dependent memory [46–48].

We began by assessing the effects of lithium on hippocampal neurogenesis in *Bbs1*^*M390R/M390R*^ and control mice. Young adult mice were treated with lithium or vehicle (water) for two weeks, and brain tissues were harvested and stained for BrdU and Doublecortin (Fig 6A and 6B). Rationale for the delivery, dosage and duration are based on previous literature explained in the methods section. Compared to vehicle treated *Bbs1*^*M390R/M390R*^ mice, lithium treatment led to a 99% increase in the number of new neural cells and a 153% increase in the number of new neurons in the dentate gyrus of the hippocampus (Fig 6C and 6D).

### Lithium treatment rescued long-term context fear conditioning in *Bbs1*^M390R/M390R^ mice

We hypothesized that enhancing hippocampal neurogenesis using lithium treatment would rescue context, but not cue fear conditioning, which is not hippocampus dependent (Fig 6D) [28]. To test the effects of lithium on fear conditioning, 4–5 week old mice were administered lithium or vehicle for two weeks (Fig 6E). The delivery, dosage and duration of lithium for fear conditioning was determined based on our previous results showing increased hippocampal neurogenesis. The mice underwent fear conditioning using the three-day paradigm (Fig 6E–6G).

As hypothesized, lithium treatment rescued the long-term context fear conditioning and not long-term cue fear conditioning in *Bbs1*^*M390R/M390R*^ mice (Fig 6G). However, our results of lithium increasing hippocampal neurogenesis and long-term context fear conditioning are correlational. Further research is needed to establish whether the increased hippocampal neurogenesis results in the improvement in long-term context fear conditioning. Overall, our results indicate that *Bbs1* gene play an important role in mediating hippocampal neurogenesis and long-term context fear conditioning, and that lithium can modulate these processes in *Bbs1*^*M390R/M390R*^ mice.

## Discussion

Intellectual disability (ID) is the most common neurodevelopmental disorder [1]. ID has limited pharmacological treatments, which is attributed to a limited understanding of the mechanisms involved. A reason for the lack of mechanistic understanding is due to a lack of mouse models of ID. To overcome this hurdle, we explored the use of a mouse model of a syndromic intellectual disability, Bardet-Biedl Syndrome (BBS), for the study of ID. BBS mouse models also provide a means to explore the association of ciliary proteins, specifically BBS proteins, to learning and memory.

We show that BBS mice have impaired context fear conditioning, indicating that BBS genes play a critical role in long-term memory. Our studies elucidate the spatial and temporal role of BBS gene function in fear memory. Using a conditional *Bbs1* knockout mouse model, we demonstrate that BBS1 in the forebrain plays an important role in long-term fear memory. These findings are consistent with prior reports that cilia in the forebrain are involved in long-term fear memory[49]. In addition, the use of a novel tamoxifen inducible *Bbs8* knockout mouse model demonstrate that BBS gene function is critical during the post-natal consolidation of long-term fear memory.

Our work is in contrast to previous work using *Bbs4* knockout mice to study the role of BBS4 in fear conditioning, which gave inconsistent results [34,35]. This is partially explained by the use of different mouse strains and testing parameters. These studies used *Bbs4* null mice on C57BL/6 [35] or FVB/NJ[34] backgrounds. In addition, these studies have noted a sex difference in BBS mice (*Bbs4* knockout mice) with respect to fear conditioning, which we do not observe [35]. The discrepancies in findings may be due to differences in mouse strains or study design. Our study primarily used strains 129/SvEv and C57BL/6. We used a strong learning paradigm with five pairings of shocks, compared to three shock pairings[35] or two shock pairings[34]. In addition, *Bbs4* knockout mice were evaluated in the previous study compared to *Bbs1*^*M390R/M390R*^ mice and *Bbs8* knockout mice in our current study. Although BBS4, BBS1 and BBS8 are all components of the BBSome, it is possible that these proteins could have unique properties on fear memory.

Since BBS is a pleiotropic disorder, there are other factors that could explain the context fear conditioning impairment observed in mouse models of BBS. The *Bbs1*^*M390R/M390R*^ mice have visual deficits [22], olfactory deficits [50], obesity [22] and hydrocephalus, which could globally affect fear conditioning. In order to control for these phenotypes, we used young adult mice prior to the onset of obesity and blindness. In addition, our BBS1 conditional knockout mice are not blind nor obese and BBS8 conditional knockout mice do not have hydrocephalus [20,51], yet both models have impaired long-term context fear conditioning. We were not able to account for the olfactory deficit as a confounding factor. However, if these phenotypes underlie the observed fear conditioning deficits, mice would also display short-term (immediate) fear conditioning deficits in addition to long-term deficits, which we do not observe. Therefore, we conclude that the fear learning deficits observed are a primary phenotype due to the absence of BBS gene function.

Other mouse models demonstrate fear memory deficits similar to those we report in this study. For example, long-term context fear conditioning, but not short-term context fear conditioning, has been reported in mice with absent neuronal nitric oxide synthase [32], mice with inhibited protein synthesis [31], and in mice with PKA [30,31] or MAP Kinase deficiencies [31]. While these mouse models have impaired long-term potentiation (LTP) in CA1 of the hippocampus, there are mouse models with impaired memory that have normal LTP [49,52] as is the case with our *Bbs1*^*M390R/M390R*^ mice.

The decreased hippocampal neurogenesis in *Bbs1*^*M390R/M390R*^ mice is a novel finding that can explain the impaired fear context memory. Hippocampal neurogenesis is involved in hippocampus dependent learning, such as context fear conditioning. Impaired context fear conditioning has been reported in mice with genetic suppression of proliferation of GFAP expressing cells [53,54] and Nestin expressing cells [55,56]. Impaired fear context long-term memory was also seen in mice with suppressed hippocampal neurogenesis through irradiation of the head [57] and ganciclovir treated mice [53], supporting our results using BBS mouse models.

We speculate that BBS proteins affect hippocampal neurogenesis because BBS is involved in ciliary receptor trafficking of the Smoothened Receptor [14,58,59], which is involved in in SHH signaling. SHH signaling is mediated by primary cilia [43]. Primary cilia are particularly enriched in the hippocampus [60]. Furthermore, SHH signaling has a proliferative effect on adult hippocampal progenitors *in vitro and in vivo* [61]. In addition, both primary cilia and smoothened receptors (hedgehog signaling) are required by adult neural stem cells [62]. The role of BBS in hippocampal proliferation may also be due to their involvement in tyrosine receptor kinase B (TrkB) receptor signaling [18]. Brain derived neurotrophic factor (BDNF) has been shown to increase neurogenesis through TrkB receptors [63,64].

We investigated lithium as a treatment for the memory and neural deficits of *Bbs1*^*M390R/M390R*^ mice. Lithium has been shown to improve learning and memory tasks in mouse models of cognitive disease including Fragile X syndrome[65], Down syndrome[48], and Alzheimer disease[46]. Lithium has also been shown to increase hippocampal neurogenesis [46,48,66,67]. While lithium treatment of *Bbs1*^*M390R/M390R*^ mice produced a robust effect on memory performance, lithium treatment produced a more modest response in hippocampal proliferation and neurogenesis. This suggests that a modest change in hippocampal proliferation and neurogenesis can produce a profound effect on memory. Our results encourage the study of an FDA approved drug, lithium, for treating ID in BBS patients.

Recent clinical studies lend support to the concept of using lithium for the treatment of ID. A Danish study shows a correlation between lower incidence of dementia and long-term exposure to lithium in drinking water[68]. In addition, a study in China showed that low-dose lithium treatment improved cognitive performance in children with ID without major side effects[69].

It is possible that lithium has other neural effects contributing to the rescue of context fear conditioning. For example, lithium has been reported to alter dendritic spine density in the hippocampus [70] and to improve olfaction in mouse models of olfactory impairment [71,72]. In addition, lithium has been reported to increase the length of cilia in cultured neurons[73]. However, this effect is unlikely to explain lithium’s memory improvement selectively in BBS mice since there is no difference in the length of cilia in cultured neurons from BBS mice and control mice [74].

Further research is needed to explore factors involved in decreased hippocampal neurogenesis in *Bbs1*^*M390R/M390R*^ mice. In our young adult mouse study, we observe a decrease in the number of BrdU+/Doublecortin+ cells in the hippocampus, indicating decreased neurogenesis. However, the apparent decrease in neurogenesis could be due to decreased proliferation, decreased survival and/or decreased differentiation. BBS proteins are involved in the function of primary cilia, and primary cilia are involved in proliferation, differentiation [75] and survival[76].

There are alternative explanations for the cause of the impaired learning and memory in BBS mouse models. BBS proteins traffic other ciliary receptors that are involved in learning and memory. *Bbs4* knockout mice [17] and *Bbs7* knockout mice [77] accumulate dopamine 1 (D1) receptors in cilia. D1 receptors are involved in learning and memory[78,79]. BBS proteins are also involved in trafficking of the somatostatin receptor 3 (SSTR3) [58] and melanin concentrating hormone receptor 1 (MCHR1) [16], both of which are involved in learning and memory [80,81]. Therefore, mislocalization of ciliary receptors for learning and memory in BBS mice could explain the impaired fear conditioning.

Another explanation for the impaired fear conditioning observed in BBS mice is altered dendrites and synapses. Changes in cilia function can alter dendritic organization and synaptic signaling. Arborization of dendrites are dependent on primary cilia [82] and primary cilia signaling is required for synaptic connection[83]. Haq et al. showed that *Bbs4* knockout mice and *Bbs5* knockout mice have moderately decreased dendritic spine density and decreased dendritic length compared to control mice. However, the *Bbs1*^*M390R/M390R*^ mice display a mild decrease in dendritic spine density and no difference in dendritic length compared to control mice. We observed robust fear conditioning deficits in *Bbs1*^*M390R/M390R*^ mice, thus it is unlikely that the altered dendrites is the main cause of the memory impairment.

Our mouse model of a ciliopathy with ID robustly presents with impaired fear memory in context fear conditioning and decreased neurogenesis. Our mouse model of BBS presents similarly to the mouse model of Fragile X Syndrome [84]. Fragile X syndrome is one of the most commonly inherited disorders for intellectual disability [85], and has recently been found to have defective cilia [86]. Further investigation is needed to determine whether there are other phenotypes that would further support BBS mice as a good model to study Intellectual Disability. Overall, the findings presented here support the use of BBS mice as a model for ID and support the use of pro-neurogenic treatments as a possible treatment for ID.

## Methods

### Ethics statement

This research was conducted in strict accordance to the Guide for the Care and Use of Laboratory Animals, 8^th^ edition, from the National Research Council. All mice were handled based on approved Institutional Animal Care and Use Committee (IACUC) protocols (#5061426 and #8072147) at the University of Iowa. Animals were housed in facilities, maintained by the Office of Animal Resources that adhere to IACUC recommendations. Mice were euthanized either by anesthesia induced by I.P injection of ketamine/xylazine followed by transcardiac perfusion, or carbon dioxide inhalation followed by cervical dislocation. Every effort was made to minimize suffering in the mice, and humane endpoints were stringently observed.

### Animals

All mice were group housed on a set 12 hr light-dark cycle and given standard chow (LM-485; Teklab, Madison, WI, USA) and water ad libitum. Mice were generated at the University of Iowa Carver College of Medicine and all experiments were performed in accordance with the Institute for Animal Care and Use Committee at the University of Iowa. For all testing, we used young adult mice (1.5–3 month old mice), unless otherwise noted. The ages of mice were chosen to keep the weight and visual processing differences between *Bbs1*^*M390R/M390R*^ and control mice to a minimal [22]. All testing was conducted during the light cycle, unless otherwise noted.

We used several strains of mice as listed below. Control mice were of the same genetic strains as the mice with which they were compared. We used male and female mice on a pure 129/SvEv genetic background for *Bbs1*^*M390R/M390R*^ mice and littermate controls (*Bbs1*^*+/+*^ and *Bbs1*^*M390R/+*^). Heterozygote mice (*Bbs1*^*M390R/+*^) do not exhibit BBS phenotypes [22,47], and are not significantly different in fear conditioning compared to *Bbs1*^*+/+*^ mice (S3 Fig). To generate mice with preferential *Bbs1* deletion in the forebrain, we crossed *Bbs1*^*flox/flox*^ mice (129/SvEv) [47] with *Emx1-Cre* knock-in mice (C57BL/6) (Jackson Laboratory, #005628). To verify forebrain *Cre* expression, we crossed *Emx1-Cre* knock-in mice with the Ai9 *Cre* reporter line *Gt(ROSA)26Sor*^*tm9(CAG-tdTomato)Hze*^ (C57BL/6) (Jackson Laboratory #007909). We also used conditional *Bbs8* knockout mice (*Bbs8*^flox/flox^, C57BL/6) [36] crossed with tamoxifen-inducible *Cre* recombinase mice, B6.Cg-*Ndor1*^*Tg(UBC-cre/ERT2)1Ejb*^/2J (Jackson Laboratory #008085).

### Tamoxifen-inducible excision of *Bbs8*

We postnatally excised *Bbs8* according to previously described procedures [36]. To induce *Cre* expression, *Bbs8*^flox/flox^ and *Bbs8*^flox/-^; *UBC-Cre*^*ERT2*^+ mice were injected subcutaneously with 40 μL of tamoxifen (15 mg/mL in corn oil) on three separate days (P9, P12, and P15). *Bbs8*^flox/flox^ and *Bbs8*^flox/-^; *UBC-Cre*^*ERT2*^- mice injected with tamoxifen were the littermate controls. We assessed excision efficiency as previously described [36]. The *Bbs8* tamoxifen inducible knockout mice (*Bbs8*^flox/flox^ and *Bbs8*^flox/-^; *UBC-Cre*^*ERT2*^+) that were determined to have less than 90% excision were excluded from the research study. This was decided as an exclusion criterion prior to conducting the study. No other mice were excluded from the research study.

### Behavioral testing

All behavioral testing was conducted during the light cycle, unless otherwise noted.

Delay Fear Conditioning: For fear conditioning, mice were placed in a fear conditioning chamber with near-infrared video. Freezing was scored with the VideoFreeze software (Med Associates, St. Albans, VT, USA). Fear conditioning can distinguish short-term context memory from long-term context memory based on when context fear conditioning is tested (short-term is 1 hour after conditioning, and long-term is ≥24 hours after conditioning) [30–32]. A 3-day protocol was used to assess both long-term cue and contextual fear conditioning.

- On the first day of fear conditioning, a 20-second tone (75 dB) was played, which co-terminated with a 1-second foot shock (0.75 mA). The tone-shock pairings occurred five times, with the shocks at 3:20m, 5:40m, 8m, 10:20m and 12:40m. For the acquisition curve figure, the freezing data was reported as the percent time the mouse was immobile for each one-minute bout. In addition, the training in day 1 fear conditioning was measured as:
  ○ Immediate fear conditioning = freezing time (%) just after conditioning (last minute)—the freezing time (%) just before conditioning (first three minutes)
- On the second day, to test cue fear conditioning, mice were tested in a novel context in which floor texture, odor, and shape of the chamber had been altered. After 3 minutes in the chamber, a 3-minute tone (75 dB) was delivered, followed by an additional 4 minutes without the tone. The cue fear conditioning was measured as:
  ○ Cue fear conditioning = freezing time (%) during the tone on day 2 –freezing time (%) before the tone on day 2
- On the third day, to test contextual fear conditioning, the chamber was set back to the original training context. Mice were place in the chamber for 5 minutes. The context fear conditioning was measured as:
  ○ Context fear conditioning = freezing time (%) on day 3—freezing time (%) just before conditioning (first three minutes of day 1).

One day fear conditioning: The acquisition protocol for three-day fear conditioning was used for the one day fear conditioning protocol. After the acquisition phase, mice were placed back into their home cage. One hour after the fear conditioning, mice were placed back into the original training chamber, and recorded for five minutes. The short-term context fear conditioning was measured as the difference of the freezing time (%) just before conditioning (first three minutes of day 1) and during the context on day 1 (one hour after fear conditioning).

Preyer Reflex: The Preyer reflex is the startle response to auditory stimuli. Mice were given an auditory stimulus (hand clap) in their home cage. A positive sign was noted if the mouse had a rapid movement of the whole body after the auditory stimulus.

Circling Behavior: Circling behavior is noted in animal models of deafness [87]. Mice were observed for 5 minutes in their home cage for circling behavior. A positive circling behavior was noted if the mouse tightly circled around itself more than two times.

### Auditory brainstem response

The auditory brainstem response (ABR) test provides information about the auditory sensitivity of the subject. The ABR test was conducted on 2-month old control mice (n = 3) and *Bbs1*^*M390R/M390R*^ mice (n = 4). The experimenter was masked to the genotype. The ABR test were conducted as previously described [88]. Briefly, clicks and tone-bursts were delivered to the testing ear through a plastic acoustic tube in a sound attenuated room. ABRs were measured using an Etymotic Research ER10B+ probe microphone (Etymotic Research, Elk Grove, IL, USA) coupled to two Tucker-Davis Technologies MF1 multi-field magnetic speakers (Tucker-Davis Technologies, Alachua, FL, USA). Click and tone-burst stimuli were presented and recorded using custom software running on a PC connected to a 24-bit external sound card (Motu UltraLite mk3, Cambridge MA, USA). A custom-built differential amplifier with a gain of 1,000 dB amplified acoustic ABR responses. Output was passed through 6-pole Butterworth high-pass (100 Hz) and low-pass (3 kHz) filters and then to a 16-bit analog-to-digital converter (100,000 sample/s). The tone bursts were 3 ms in length, in addition to 1 ms onset and offset ramps (raised cosine shape) centered at 4, 8, 16, 24, and 32 kHz. Responses were recorded using standard signal-averaging techniques for 500 or 1000 sweeps. Hearing thresholds (db SPL) were determined by decreasing the sound intensity by 5 and/or 10 db decrements and recording the lowest sound intensity level resulting in a recognizable and reproducible ABR response wave pattern. Maximum ABR thresholds were capped at 100 db SPL.

### BrdU injections

For early postnatal time points, mice were injected intraperitoneally with 300mg/kg Bromodeoxyuridine (BrdU, Sigma, St. Louis, Missouri) and sacrificed 4 hours after the injection. For later postnatal time points, mice were injected intraperitoneally with 50mg/kg BrdU twice a day for five days, and sacrificed ten days later.

### Lithium treatment

We treated mice with 45mM lithium chloride (Sigma) in drinking water starting at four to five weeks of age for 2 weeks. Delivery of lithium through drinking water was based on previous BBS mouse studies [47]. The lithium dosage was determined in previous studies that showed that 50mM of lithium for two weeks is the highest dosage that results in serum levels therapeutic to humans without adverse effects in the mice [89]. We chose the duration of the lithium based on the duration reported to enhance hippocampal neurogenesis [67]. Mice were group-housed for lithium treatment and group-housed for vehicle (water) treatment. Littermate controls were group-housed.

### Tissue collections and histology

For early postnatal tissue, fresh brain tissues were collected and embedded in Optimal Cutting Temperature compound (OCT, Sakura). Eight μm sections were cut on a cryostat. For late postnatal time points, mice were anesthetized by intraperitoneal injection of Ketamine (17.5 mg/cc)/Xylazine (2.5 mg/cc) at 100 μL/20 gram body weight, and transcardially perfused with 4% paraformaldehyde in phosphate-buffered saline (PBS). The brain and eyes were removed and post-fixed overnight at 4°C with 4% paraformaldehyde in PBS, followed by more than 24 hours of immersion in 30% sucrose in PBS. Tissues were then embedded in OCT (Sakura). 20 μm sections were cut on a cryostat.

### Immunohistochemistry

Tissue sections were directly placed on positively charged microscope slides (Globe Scientific). Tissue sections were fluorescently immunostained for the following markers: anti-tdtomato: 1∶500 rabbit polyclonal anti-DsRed (Takara), immature neuronal marker anti-Doublecortin (1:500, abcam) proliferative marker anti-BrdU (1:200, Abcam). Tissue sections were then stained with a fluorescently tagged secondary antibodies (488 dye, 568 dye, 633 dye, ThermoFisher Scientific). Antigen retrieval was used for neuronal markers (50 mM Tris HCl, 45 minutes at 80°C) and BrdU marker (2N HCl, 0.1% triton, 30 minutes in 37°C)

In preparation for staining, slides with sections were placed in 50 mM Tris HCl for 45 minutes at 80°C. After slides were cooled, slides were incubated in 2N HCl (0.1% Triton) at 37°C for 30 minutes and rinsed in 0.1 M boric acid (pH 8.5) at room temperature for 10 minutes. Sections were then rinsed in PBST (0.2% Triton X-100/ PBS), blocked for one hour with block solution (2% bovine serum in PBS/0.1% Triton X-100), and incubated overnight with anti-BrdU antibody and anti-Hu antibody in serum solution (1% bovine serum in PBS/0.1% Triton X-100) at 4°C. Sections were washed in PBST, incubated with secondary antibodies in serum solution at RT for 2 hours, washed in PBST, counterstained with DAPI, and cover slipped with antifade mounting medium from Vector Laboratories (H-1900).

### Cell quantification

Tissue sections used for BrdU quantification were imaged using an inverted fluorescent microscope (Olympus IX71). Exposure was kept constant for each channel within experiments. Four to six representative dentate gryi from coronal sections were counted per mouse subject. The number of BrdU+ cells were counted within the dentate gyrus of a 20x image (Fig 5D–5F). BrdU is a maker for proliferating cells. The BrdU+ cell counts were standardized to the volume of dentate gyrus tissue in the image (mm^3^). Volume was determined by measuring the area of the dentate gyrus on ImageJ and multiplying the area by the thickness of the tissue. For analysis of neurogenesis, Doublecortin+BrdU+ and BrdU+ cell within the dentate gyrus were counted. Doublecortin is a marker for immature neurons [45]. The tissue sections used for quantification were imaged using confocal microscopy (SP8 confocal microscope, Leica). To determine the frequency of BrdU+ cells expressing DCX, dual fluorescence-labeled sections were examined by confocal microscopy using a 20x objective (Fig 6B). Sections were scored for single or double labeling by manual examination of optical slices. Cells were manually counted for double labeling when DCX labeling was unambiguously associated with a BrdU+ nucleus. Cells were spot-checked in all three dimensions by Z-stack using a 20x objective. In all cases, the observer was masked to the treatment and genotypes.

### Comprehensive lab animal monitoring system

For analysis of whole animal activity levels and sleep behavior, *Bbs1*^*M390R/M390R*^ mice (n = 7) and control mice (n = 9) were placed in a Comprehensive Lab Animal Monitoring System (CLAMS; Columbus Instruments, Columbus, OH, USA). CLAMS is an open circuit system that directly measures various parameters over a 72 hour period including movement, sleep behavior, food intake, VO2, VCO2, and heat production. Mice were weighed before the CLAMS recording. Mice were individually housed in Plexiglas cage chambers that were kept at 24°C under a 12:12 hour light-dark cycle. The chamber had 0.6 liters of air passed per minute. Movement (activity) was measured by XY laser beam interruption, and sleep behavior was measured as minimum movement for four minutes or longer. Food consumption was monitored by electronic scales. For measuring the O2 and CO2, the gas content of the exhaust air from each chamber was compared with the gas content of the ambient air sample. The $\dot{V}O2$ and $\dot{V}\mathrm{CO}2$ measurements were normalized to mouse body weight. The following parameters were calculated as followed: RER = $\dot{V}\mathrm{CO}2$/$\dot{V}O2$, heat production = 1.232*VCO2+3.815*VO2. CLAMS were performed at the University of Iowa Fraternal Order of Eagles Diabetes Research Center Metabolic Phenotyping Core.

### Slice preparation and electrophysiology

Hippocampal slices from group-housed, naive 2-months old *Bbs1*^*M390R/M390R*^ mice (n = 4) and control mice (n = 4) were prepared as previously described [90].First, tissue brain blocks were affixed to the cutting stage, submersed in cutting solution, and transversely sectioned at 400 μm on a Vibratome 1000 Plus (Vibratome, St. Louis, MO). After bisecting into hemispheres, slices were transferred to a holding chamber containing artificial cerebrospinal fluid (aCSF). After 30 minutes, the holding chamber was removed from the water bath and held at RT (22°C) for the remainder of the experiment.

To record field excitatory post-synaptic potentials (fEPSP), aCSF-filled borosilicate electrodes (Corning #0010 glass, resistance <1 MΩ) were positioned in the stratum radiatum of area CA1. Synaptic responses were evoked by stimulation of Schaffer collaterals with bipolar tungsten electrodes (0.1 MΩ, parylene coated; World Precision Instruments, Sarasota, FL). Signals were amplified (AxoClamp 900A Amplifier, Axon Instruments, Foster City, CA), filtered at 1 kHz, digitally-sampled at 10 kHz (Axon Digidata 1440), and stored for offline analysis in Clampfit 10 (Molecular Devices, San Jose, CA).

An input-output curve (initial slope of fEPSP plotted against stimulus intensity) for assessment of basal synaptic transmission was first generated by delivering pulses of 0.2 ms duration every 15 s at increasing stimulation intensities to elicit synaptic responses. Stimulation intensity was then adjusted to yield 40–60% of the maximal fEPSP amplitude. The input/output curve is primarily used to calibrate the setting for LTP of the tissue, along with assessing the viability of the tissue.

After acquiring stable baseline responses for 15 minutes, LTP was induced by a theta-burst stimulation protocol consisting of 12 bursts of 4 pulses at 100 Hz. Synaptic responses were sampled every 15 s for 1 h after induction. For analysis, the initial slope of each fEPSP was normalized to the average baseline slope. Time-matched, normalized slopes were then averaged among slices from animals of the same genotype for comparison and plotted as an average of four consecutive responses (*i*.*e*., responses sampled over 1 minute). Slices with maximal fEPSPs of less than 0.5 mV, disproportionately large fiber volleys, substantial changes in fiber volley amplitude during LTP recordings, or unstable synaptic responses during baseline or LTP recordings were excluded.

### Statistical analysis

Statistical analyses were performed using GraphPad Prism 8.0 (GraphPad Software, San Diego, CA). Data used for statistical analysis are in the S1 Data file. For comparison of two groups, we ran a two-tailed Welch’s t-test. The Welch’s t-test is recommended over the Student t-test because Welch’s t-test performs better when the sample sizes and variances are unequal between groups, and gives similar results when sample sizes and variances are equal [91,92]. Preliminary tests of equality of variances to determine t-tests are not recommended since it impairs the validity of the Welch’s t-test [93]. For data that appear skewed, we also ran a Mann-Whitney-Wilcoxon Test. For multiple comparisons, we ran multiple t-tests (analyzed each row individually and did not assume consistent standard deviations) corrected for multiple comparisons using the Holm-Sidak method. A two-way ANOVA was used for comparisons of multiple groups with two different independent variables. We then ran a Sidak post-hoc analysis for the relevant data. A three-way ANOVA was used for comparison of multiple groups with three different independent variables. Graphs were generated on GraphPad Prism 8.0. Data are presented as mean with the error bars indicating standard error of means (unless otherwise noted).

## Supporting information

S1 Fig Fear conditioning of Wild-type mice (*Bbs1*^*+/+*^) and Heterozygote mice (*Bbs1*^*+/M390R*^). The data is collated from Figs 1 and 6. The immediate fear conditioning indicates training to the day 1 fear conditioning. The immediate fear conditioning was measured as the freezing time (%) increase of the freezing time (%) just after conditioning (last minute) to the freezing time (%) just before conditioning (first three minutes). The 24 hr fear conditioning represents cue fear conditioning, and was measured as the freezing time (%) increase of the freezing time (%) during the tone (cue, day 2) to the freezing time (%) before the tone (cue, day 2). The 48 hr fear conditioning represents context fear conditioning, and was measured as the freezing time (%) increase of the freezing time (%) during the context on day 3 to the freezing time (%) just before conditioning (first three minutes of day 1). A.) The immediate fear conditioning was not significantly different between the Wild-type mice (n = 7) and Heterozygote mice (n = 22) (Welch’s t-test, P = 0.494432). The 24 hr fear conditioning (cue) was not significantly different between the Wild-type mice (n = 7) and Heterozygote mice (n = 22) (Welch’s t-test, P = 0.814487). The 48 hr fear conditioning (context) between the Wild-type mice (n = 7) and Heterozygote mice (n = 22) was not significantly different (Welch’s t-test, P = 0.746392). hr = hours, ns = not significant.
(TIF)

S2 Fig No sex effects on fear conditioning in control and *Bbs1*^*M390R/M390R*^ mice. All data in S2 Fig is from the same data pool as Fig 1. The immediate fear conditioning indicates training to the day 1 fear conditioning. The immediate fear conditioning was measured as the freezing time (%) increase of the freezing time (%) just after conditioning (last minute) to the freezing time (%) just before conditioning (first three minutes). The 24 hr fear conditioning represents cue fear conditioning, and was measured as the freezing time (%) increase of the freezing time (%) during the tone (cue, day 2) to the freezing time (%) before the tone (cue, day 2). The 48 hr fear conditioning represents context fear conditioning, and was measured as the freezing time (%) increase of the freezing time (%) during the context on day 3 to the freezing time (%) just before conditioning (first three minutes of day 1). A.) The immediate fear conditioning was not significantly different between the Male control mice (n = 7) and Female control mice (n = 9) (Welch’s t-test, P = 0.251805). The 24 hr fear conditioning (cue) was not significantly different between the Male control mice (n = 7) and Female control mice (n = 9) (Welch’s t-test, P = 0.610210). The 48 hr fear conditioning (context) between the Male control mice (n = 7) and Female control mice (n = 9) was not significantly different (Welch’s t-test, P = 0.877612). B.) The immediate fear conditioning was not significantly different between the Male *Bbs1*^*M390R/M390R*^ mice (n = 7) and Female *Bbs1*^*M390R/M390R*^ mice (n = 9) (Welch’s t-test, P = 0.470202). The 24 hr fear conditioning (cue) was not significantly different between the Male *Bbs1*^*M390R/M390R*^ mice (n = 7) and Female *Bbs1*^*M390R/M390R*^ mice (n = 9) (Welch’s t-test, P = 0.476866). The 48 hr fear conditioning (context) between the Male *Bbs1*^*M390R/M390R*^ mice (n = 7) and Female *Bbs1*^*M390R/M390R*^ mice (n = 9) was not significantly different (Welch’s t-test, P = 0.963440). control mice = *Bbs1*^*M390R/+*^ mice, M390R mice = *Bbs1*^*M390R/M390R*^ mice, hr = hours, ns = not significant.
(TIF)

S3 Fig Hearing and behavioral assessments of *Bbs1*^*M390R/M390R*^ mice. A.) Graph of Auditory Brainstem Response. The threshold of the Auditory Brainstem Response for the control mice (n = 3) and *Bbs1*^*M390R/M390R*^ mice (n = 4) were not significantly different in clicks (P = 0.758), 4khz (P = 0.813), 8khz (P = 0.922), 16khz (P = 0.813), 24khz (P = 0.922), and 32khz (P = 0.813). Comparisons were analyzed using multiple t-test (without assumption of consist standard deviation), corrected with the Holm-Sidak method. B.) Table of behavioral hearing test. Both the control mice (n = 7, female n = 2) and *Bbs1*^*M390/M390R*^ mice (n = 9, female n = 4) had intact Preyer reflex and no circling behavior. C.) Maximum motion index to the first shock in fear conditioning (Shock Reactivity). The control mice (n = 13) and the *Bbs1*^*M390R/M390R*^ mice (n = 13) were not significantly different in shock reactivity (Welch’s t-test, P = 0.672). D.) The control mice (n = 9) and the *Bbs1*^*M390R/M390R*^ mice (n = 7) were not significantly different in total activity at Light Cycle (P = 0.830) or Dark Cycle (0.908). Comparisons were analyzed using multiple t-test (without assumption of consist standard deviation), corrected with the Holm-Sidak method. E.) The control mice (n = 9) and the *Bbs1*^*M390R/M390R*^ mice (n = 7) did were not significantly different for sleep behavior at Light Cycle (P = 0.608) or Dark Cycle (P = 0.608). Comparisons were analyzed using multiple t-test (without assumption of consist standard deviation), corrected with the Holm-Sidak method. control = *Bbs1*^*M390R/+*^ mice, M390R = *Bbs1*^*M390R/M390R*^ mice, ABR = Auditory Brainstem Response, ns = not significant.
(TIF)

S4 Fig *Emx1-Cre* mice. A.) Preferential Cre expression in the forebrain. Tissue sections from mice with either Ai9 TdTomato or Ai9TdTomato and *Emx1*-Cre. Ai9 mice do not have any ectopic expression of red fluorescent protein in the brain and retina. Ai9 mice with *Emx1*-Cre have preferential expression of red fluorescent protein in the forebrain, and not in the eye. Ai9 Tdtomato staining (red) and DAPI nuclear staining (blue). The white line on the brain sections indicates 0.5mm, and the white line on the eye sections indicates 1.0mm. B.) Preferential Cre expression in the forebrain. Brain tissue from mice with either Ai9 TdTomato or Ai9TdTomato and *Emx1*-Cre. Ai9 mice do not have any ectopic expression of red fluorescent protein. Ai9 mice with *Emx1*-Cre have preferential expression of red fluorescent protein in the cortex and olfactory bulb. C.) DNA gel. Excision band preferentially seen in the forebrain of *Bbs1*^flox/flox^; *Emx1-Cre*+ mice. F = Forebrain, H = Hindbrain, 1, 2 = *Emx1-Cre*+ mice, 3, 4 = *Bbs1*^flox/flox^; *Emx1-Cre*+ mice Cer = Cerebellum, Ctx = Cortex, Hyp = Hypothalamus, Tha = Thalamus, Amy = Amygdala, Hip = Hippocampus, OB = Olfactory Bulb, Ret = Retina, VB = Vitreous Body.
(TIF)

S1 Data Excel of raw data file of all relevant figures. This includes data for Figs 1–6 and S1–S3.
(XLSX)
