# Supplementary figures and images for: A mouse model of Bardet-Biedl Syndrome has impaired fear memory, which is rescued by lithium treatment
Source: PLoS Genet. 2021 Apr 22;17(4):e1009484. doi: 10.1371/journal.pgen.1009484 (PMC8061871; doi:10.1371/journal.pgen.1009484)

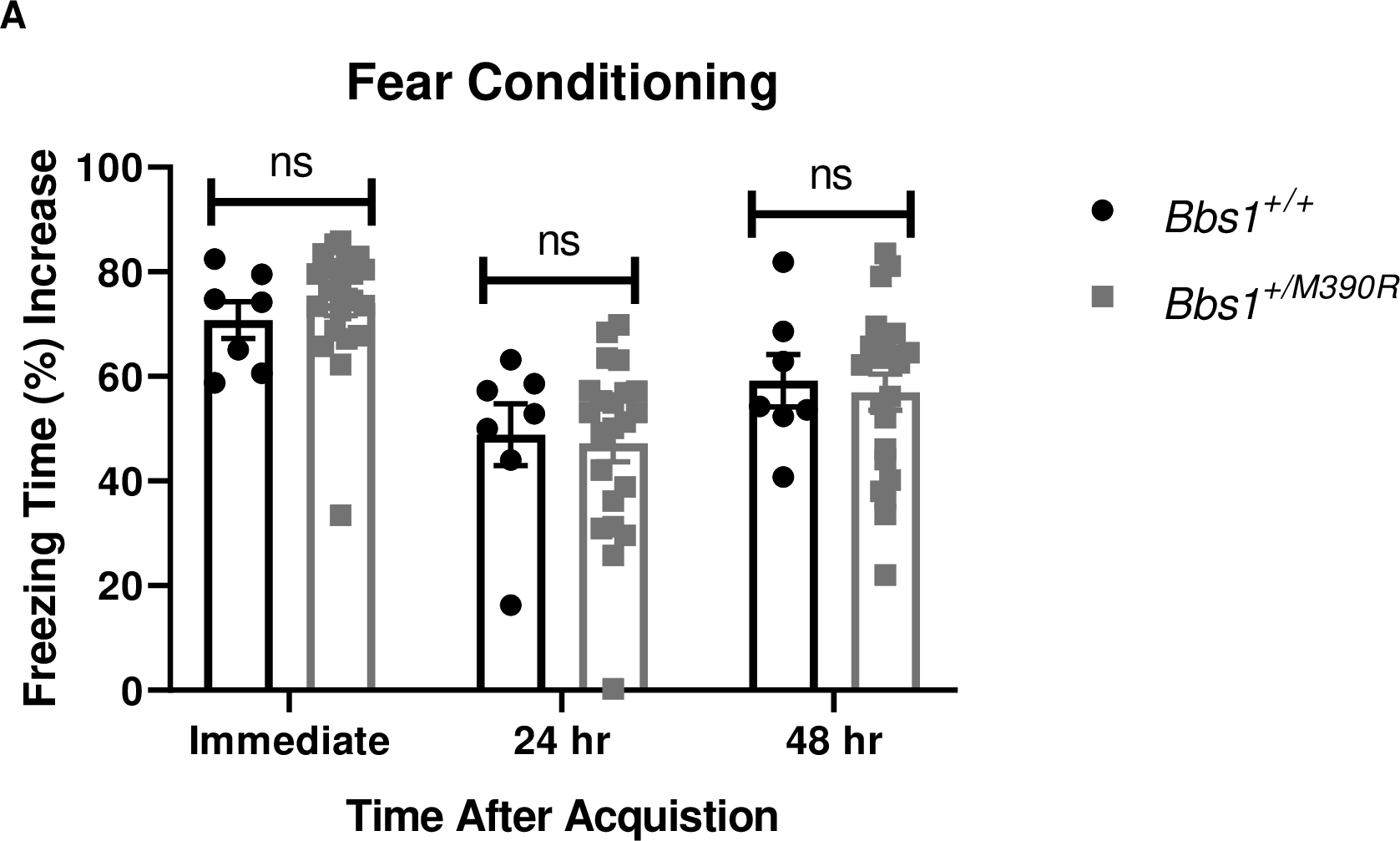

Supplement: S1 Fig — The data is collated from Figs 1 and 6. The immediate fear conditioning indicates training to the day 1 fear conditioning. The immediate fear conditioning was measured as the freezing time (%) increase of the freezing time (%) just after conditioning (last minute) to the freezing time (%) just before conditioning (first three minutes). The 24 hr fear conditioning represents cue fear conditioning, and was measured as the freezing time (%) increase of the freezing time (%) during the tone (cue, day 2) to the freezing time (%) before the tone (cue, day 2). The 48 hr fear conditioning represents context fear conditioning, and was measured as the freezing time (%) increase of the freezing time (%) during the context on day 3 to the freezing time (%) just before conditioning (first three minutes of day 1). A.) The immediate fear conditioning was not significantly different between the Wild-type mice (n = 7) and Heterozygote mice (n = 22) (Welch’s t-test, P = 0.494432). The 24 hr fear conditioning (cue) was not significantly different between the Wild-type mice (n = 7) and Heterozygote mice (n = 22) (Welch’s t-test, P = 0.814487). The 48 hr fear conditioning (context) between the Wild-type mice (n = 7) and Heterozygote mice (n = 22) was not significantly different (Welch’s t-test, P = 0.746392). hr = hours, ns = not significant. (TIF) [file pgen.1009484.s001.tif]

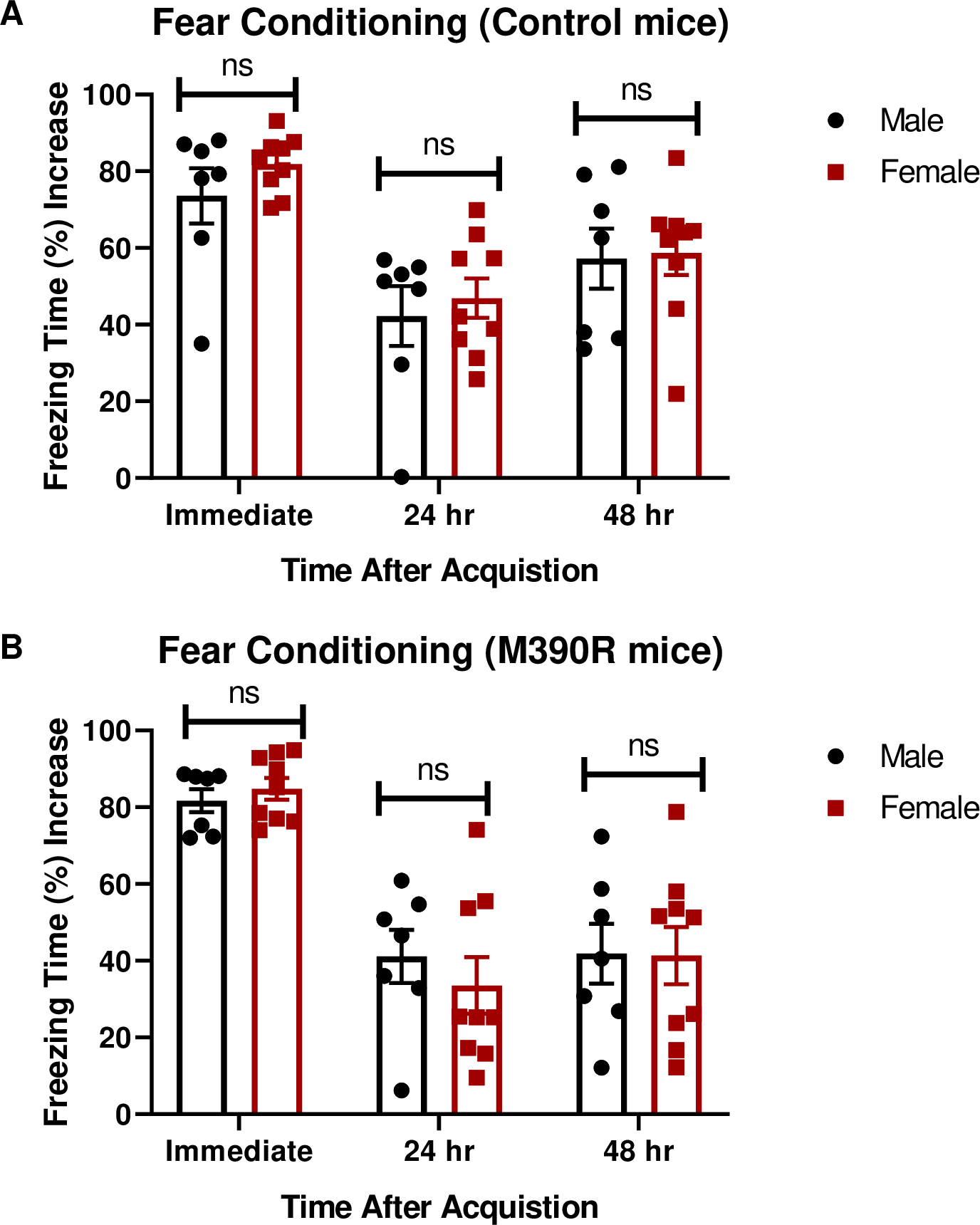

Supplement: S2 Fig — All data in S2 Fig is from the same data pool as Fig 1. The immediate fear conditioning indicates training to the day 1 fear conditioning. The immediate fear conditioning was measured as the freezing time (%) increase of the freezing time (%) just after conditioning (last minute) to the freezing time (%) just before conditioning (first three minutes). The 24 hr fear conditioning represents cue fear conditioning, and was measured as the freezing time (%) increase of the freezing time (%) during the tone (cue, day 2) to the freezing time (%) before the tone (cue, day 2). The 48 hr fear conditioning represents context fear conditioning, and was measured as the freezing time (%) increase of the freezing time (%) during the context on day 3 to the freezing time (%) just before conditioning (first three minutes of day 1). A.) The immediate fear conditioning was not significantly different between the Male control mice (n = 7) and Female control mice (n = 9) (Welch’s t-test, P = 0.251805). The 24 hr fear conditioning (cue) was not significantly different between the Male control mice (n = 7) and Female control mice (n = 9) (Welch’s t-test, P = 0.610210). The 48 hr fear conditioning (context) between the Male control mice (n = 7) and Female control mice (n = 9) was not significantly different (Welch’s t-test, P = 0.877612). B.) The immediate fear conditioning was not significantly different between the Male Bbs1M390R/M390R mice (n = 7) and Female Bbs1M390R/M390R mice (n = 9) (Welch’s t-test, P = 0.470202). The 24 hr fear conditioning (cue) was not significantly different between the Male Bbs1M390R/M390R mice (n = 7) and Female Bbs1M390R/M390R mice (n = 9) (Welch’s t-test, P = 0.476866). The 48 hr fear conditioning (context) between the Male Bbs1M390R/M390R mice (n = 7) and Female Bbs1M390R/M390R mice (n = 9) was not significantly different (Welch’s t-test, P = 0.963440). control mice = Bbs1M390R/+ mice, M390R mice = Bbs1M390R/M390R mice, hr = hours, ns = not significant. (T [file pgen.1009484.s002.tif]

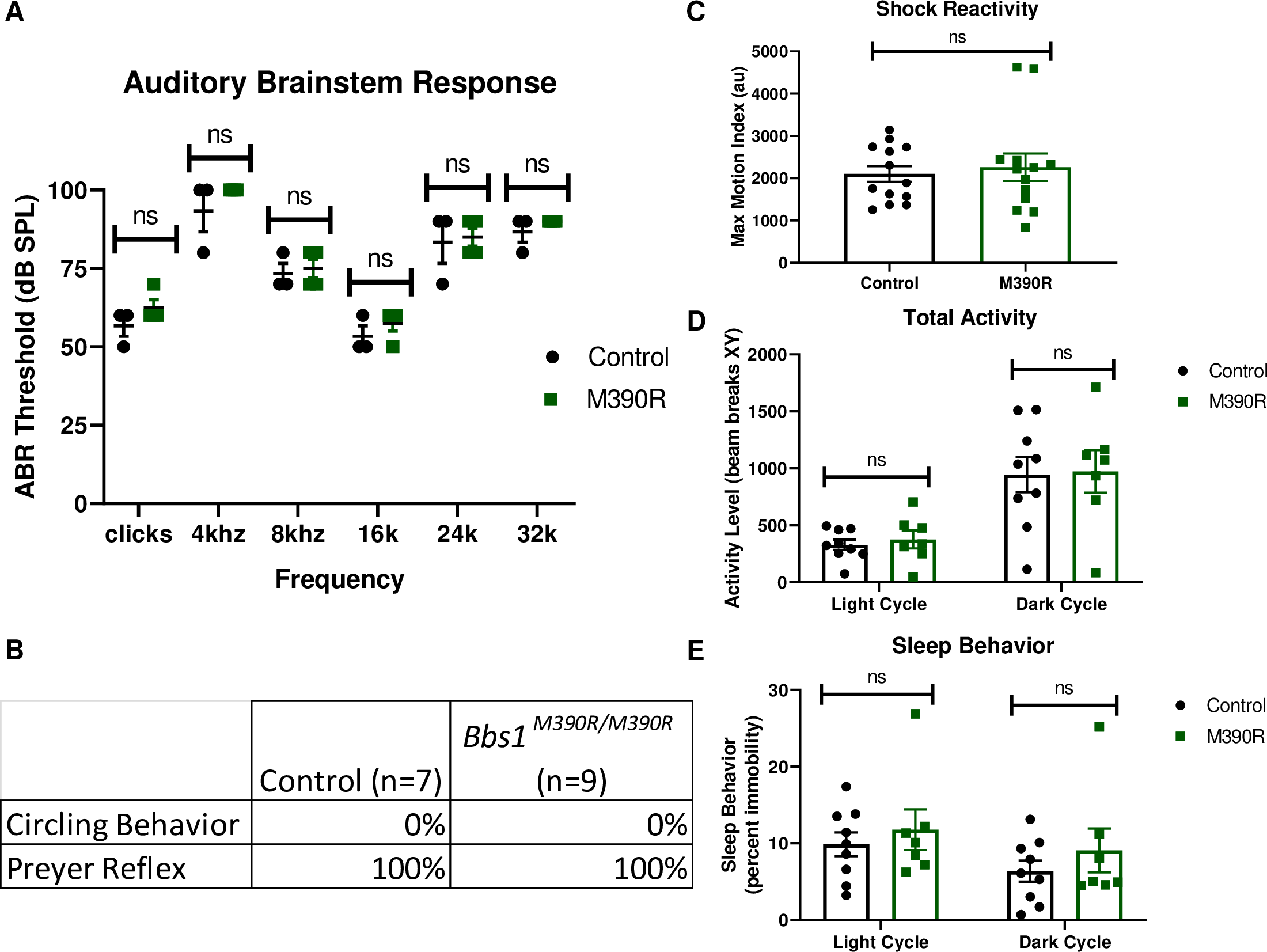

Supplement: S3 Fig — A.) Graph of Auditory Brainstem Response. The threshold of the Auditory Brainstem Response for the control mice (n = 3) and Bbs1M390R/M390R mice (n = 4) were not significantly different in clicks (P = 0.758), 4khz (P = 0.813), 8khz (P = 0.922), 16khz (P = 0.813), 24khz (P = 0.922), and 32khz (P = 0.813). Comparisons were analyzed using multiple t-test (without assumption of consist standard deviation), corrected with the Holm-Sidak method. B.) Table of behavioral hearing test. Both the control mice (n = 7, female n = 2) and Bbs1M390/M390R mice (n = 9, female n = 4) had intact Preyer reflex and no circling behavior. C.) Maximum motion index to the first shock in fear conditioning (Shock Reactivity). The control mice (n = 13) and the Bbs1M390R/M390R mice (n = 13) were not significantly different in shock reactivity (Welch’s t-test, P = 0.672). D.) The control mice (n = 9) and the Bbs1M390R/M390R mice (n = 7) were not significantly different in total activity at Light Cycle (P = 0.830) or Dark Cycle (0.908). Comparisons were analyzed using multiple t-test (without assumption of consist standard deviation), corrected with the Holm-Sidak method. E.) The control mice (n = 9) and the Bbs1M390R/M390R mice (n = 7) did were not significantly different for sleep behavior at Light Cycle (P = 0.608) or Dark Cycle (P = 0.608). Comparisons were analyzed using multiple t-test (without assumption of consist standard deviation), corrected with the Holm-Sidak method. control = Bbs1M390R/+ mice, M390R = Bbs1M390R/M390R mice, ABR = Auditory Brainstem Response, ns = not significant. (TIF) [file pgen.1009484.s003.tif]

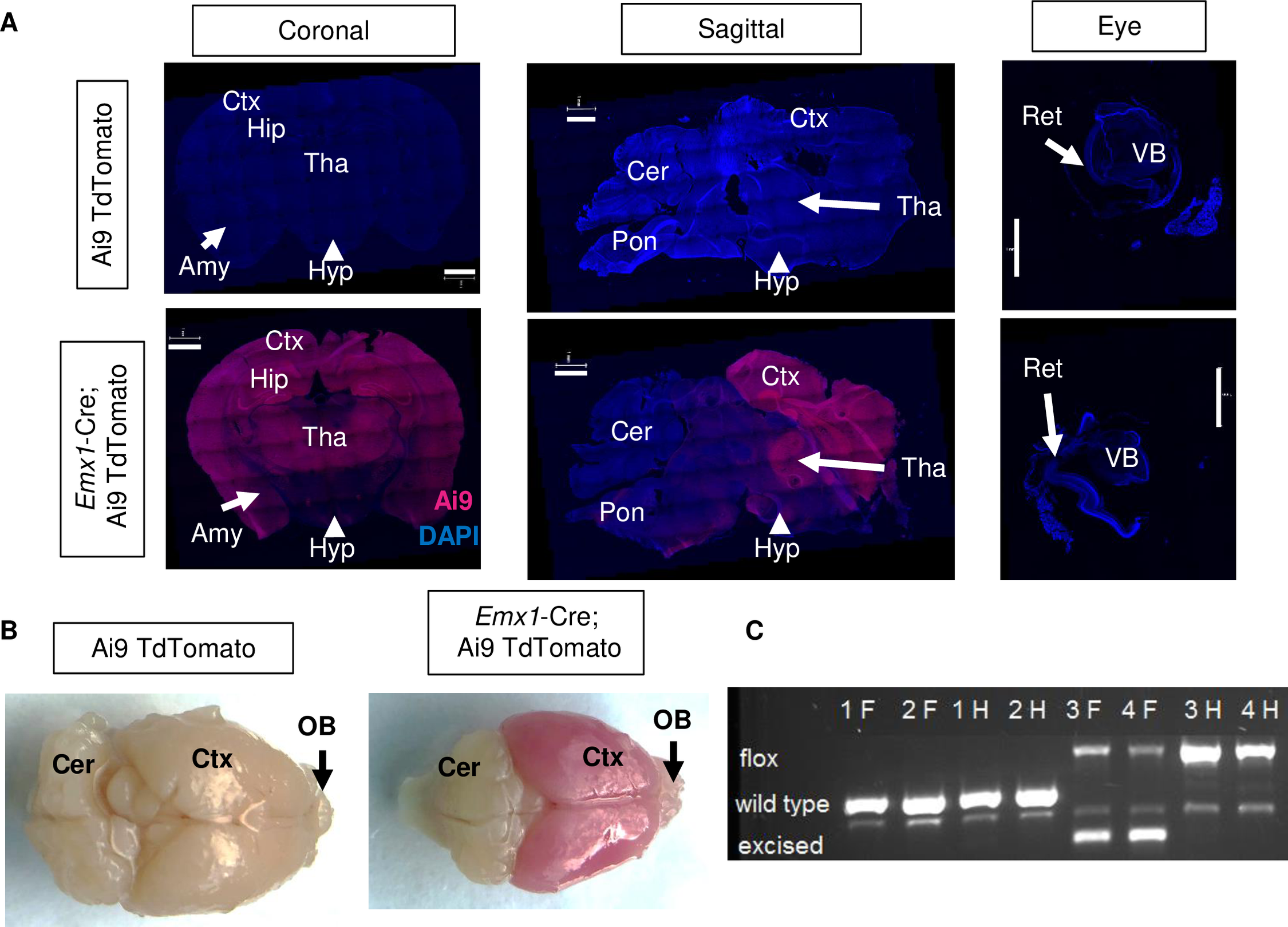

Supplement: S4 Fig — A.) Preferential Cre expression in the forebrain. Tissue sections from mice with either Ai9 TdTomato or Ai9TdTomato and Emx1-Cre. Ai9 mice do not have any ectopic expression of red fluorescent protein in the brain and retina. Ai9 mice with Emx1-Cre have preferential expression of red fluorescent protein in the forebrain, and not in the eye. Ai9 Tdtomato staining (red) and DAPI nuclear staining (blue). The white line on the brain sections indicates 0.5mm, and the white line on the eye sections indicates 1.0mm. B.) Preferential Cre expression in the forebrain. Brain tissue from mice with either Ai9 TdTomato or Ai9TdTomato and Emx1-Cre. Ai9 mice do not have any ectopic expression of red fluorescent protein. Ai9 mice with Emx1-Cre have preferential expression of red fluorescent protein in the cortex and olfactory bulb. C.) DNA gel. Excision band preferentially seen in the forebrain of Bbs1flox/flox; Emx1-Cre+ mice. F = Forebrain, H = Hindbrain, 1, 2 = Emx1-Cre+ mice, 3, 4 = Bbs1flox/flox; Emx1-Cre+ mice Cer = Cerebellum, Ctx = Cortex, Hyp = Hypothalamus, Tha = Thalamus, Amy = Amygdala, Hip = Hippocampus, OB = Olfactory Bulb, Ret = Retina, VB = Vitreous Body. (TIF) [file pgen.1009484.s004.tif]
